# In Utero Transplantation of Expanded Autologous Amniotic Fluid Stem Cells Results in Long‐Term Hematopoietic Engraftment

**DOI:** 10.1002/stem.3039

**Published:** 2019-07-29

**Authors:** Stavros P. Loukogeorgakis, Panicos Shangaris, Enrica Bertin, Chiara Franzin, Martina Piccoli, Michela Pozzobon, Sindhu Subramaniam, Alfonso Tedeschi, Aimee G. Kim, Haiying Li, Camila G. Fachin, Andre I. B. S. Dias, John D. Stratigis, Nicholas J. Ahn, Adrian J. Thrasher, Paola Bonfanti, William H. Peranteau, Anna L. David, Alan W. Flake, Paolo De Coppi

**Affiliations:** ^1^ Stem Cells and Regenerative Medicine, Great Ormond Street Institute of Child Health University College London London United Kingdom; ^2^ Center for Fetal Research The Children's Hospital of Philadelphia Philadelphia Pennsylvania USA; ^3^ Research Department of Maternal and Fetal Medicine, Institute for Women's Health University College London London United Kingdom; ^4^ Stem Cell and Regenerative Medicine Laboratory, Fondazione Instituto di Ricerca Pediatrica Città della Speranza University of Padova Padova Italy; ^5^ Department of Women's and Children's Health University of Padova Padova Italy; ^6^ Federal University of São Paulo São Paulo Brazil; ^7^ Federal University of Paraná Curitiba Brazil; ^8^ Molecular and Cellular Immunology Section, Great Ormond Street Institute of Child Health University College London London United Kingdom; ^9^ The Francis Crick Institute London United Kingdom

**Keywords:** Fetal stem cells, Autologous stem cell transplantation, Hematopoiesis, Cell culture, Transplantation tolerance

## Abstract

In utero transplantation (IUT) of hematopoietic stem cells (HSCs) has been proposed as a strategy for the prenatal treatment of congenital hematological diseases. However, levels of long‐term hematopoietic engraftment achieved in experimental IUT to date are subtherapeutic, likely due to host fetal HSCs outcompeting their bone marrow (BM)‐derived donor equivalents for space in the hematopoietic compartment. In the present study, we demonstrate that *amniotic fluid stem cells* (*AFSCs*; *c‐Kit*+/*Lin*−) have hematopoietic characteristics and, thanks to their fetal origin, favorable proliferation kinetics in vitro and in vivo, which are maintained when the cells are expanded. IUT of *autologous*/*congenic* freshly isolated or cultured AFSCs resulted in stable multilineage hematopoietic engraftment, far higher to that achieved with BM‐HSCs. Intravascular IUT of *allogenic* AFSCs was not successful as recently reported after intraperitoneal IUT. Herein, we demonstrated that this likely due to a failure of timely homing of donor cells to the host fetal thymus resulted in lack of tolerance induction and rejection. This study reveals that intravascular IUT leads to a remarkable hematopoietic engraftment of AFSCs in the setting of autologous/congenic IUT, and confirms the requirement for induction of central tolerance for allogenic IUT to be successful. Autologous, gene‐engineered, and in vitro expanded AFSCs could be used as a stem cell/gene therapy platform for the in utero treatment of inherited disorders of hematopoiesis. stem cells *2019;37:1176–1188*

**Significance Statement**

Amniotic fluid stem cells (AFSCs) can be expanded without losing their hematopoietic characteristics. In utero transplantation of allogenic AFSCs results in adaptive immune response and donor cell rejection due to failure of timely homing of donor cells to the host fetal thymus and lack of central tolerance induction. Autologous/congenic amniotic fluid stem cells can be transplanted in utero, which results in stable, multilineage, long‐term hematopoietic engraftment that is significantly higher than that achieved with bone marrow‐derived cells and could be therapeutic in many inherited disorders of hematopoiesis.

## Introduction

In utero transplantation (IUT) of hematopoietic stem cells (HSCs) is a potential nonmyeloablative strategy for the treatment of many hematologic disorders 1, 2. Engraftment of allogenic HSCs requires both induction of donor‐specific immunologic tolerance through thymic processing of donor antigen 3, 4, 5, 6 and successful competition of donor HSCs for receptive niches in the robust, nonmyeloablated fetal hematopoietic compartment 7. Failure of either component results in ultimate failure of engraftment. Experimentally, engraftment of donor cells after IUT can be accomplished across full major histocompatibility complex (MHC) barriers under specific circumstances in both murine 8, 9 and large animal models 10, 11, 12. In the murine model of allogenic IUT, we and others have shown that successful engraftment of adult bone marrow (BM) cells requires effective delivery of a relatively large dose of cells within a specific gestational age time window to achieve tolerance 13, 14, 15. On the contrary, highly enriched HSCs do not engraft even in high doses 3, suggesting that, in the BM, there are cell populations other than the HSCs required for successful antigen presentation in the fetal thymus. The developmental status of donor cells also influences engraftment with fewer fetal liver‐derived cells being required than adult BM, presumably due to the greater competitive capacity of fetal HSCs 9, 16. However, fetal liver cells are difficult to obtain, autologous transplantation would not be possible, and their isolation from aborted fetuses has raised challenging ethical questions. Although we previously documented an adaptive immune response in recipients of IUT that were nonchimeric 14, this immune response was subsequently demonstrated to be secondary to a maternal alloresponse after IUT that was passed via allo‐antibodies in breast milk to induce the adaptive response in the pups 17. In pups placed on a foster (naïve) mother after allogenic IUT, all of the recipients engraft and there is no evidence of an allo‐immune response 17.

In this context, we wished to assess the hematopoietic potential of a promising source of donor cells, the amniotic fluid stem cell (AFSC) 18, in the well‐characterized murine model of IUT. In the last few years, we have explored the hematopoietic potential of C‐Kit+ (CD117+)/Lineage− (Lin−) AFSCs 19. Freshly isolated murine AFSCs have a phenotype similar to fetal liver (FL) HSCs 20 and can engraft after postnatal transplantation into adult immunocompromised hosts 19. We have also recently demonstrated that intraperitoneal IUT of AFSCs generates multilineage hematopoietic engraftment 21. Similarly, freshly isolated CD34+ sheep AFSCs can be genetically modified overnight and are able to engraft after IUT in the hematopoietic system in an autologous setting 22. AFSCs have several potential advantages that may be salutary for IUT. They can be easily derived from routine amniocentesis, which is commonly performed for prenatal diagnosis of genetic disorders, and early second trimester collection would allow expansion in time for autologous treatment of the target disease using a stem cell/gene therapy approach 20, 23. Their fetal source and high proliferative capacity in vivo may allow them to more effectively compete with endogenous FL‐HSCs resulting in higher levels of engraftment compared with adult‐derived cells. Moreover, they can be efficiently genetically modified allowing the potential for an autologous cell‐gene therapy or gene editing approach 18, 22, 24. However, their therapeutic utility has been so far limited because their hematopoietic potential was lost after in vitro expansion using “mesenchymal‐type” culture protocols (culture in adherence with serum rich media) 18, 20. Here, we demonstrate for the first time that expansion of AFSCs on feeder layers of mitotically inactivated embryonic fibroblasts using “embryonic‐type” culture media maintains their hematopoietic characteristics and results in highly efficient engraftment of in the congenic murine model of IUT. Conversely, no engraftment was seen in the allogenic setting suggesting inadequate presentation of donor antigen in the developing/fetal thymus. These findings support the potential use of in vitro‐expanded, autologous AFSCs for the treatment of hematopoietic disorders.

## Materials and Methods

### Animals

Time‐dated C57BL/6J (B6; H2Kb+) or Balb/c (H2Kd+) pregnant mice were used as fetal IUT recipients at 14 days of gestation (E14; Fig. 2A). AFSCs were isolated at E13 from C57BL/6TgN (act‐enhanced green fluorescent protein) OsbY01 (B6‐green fluorescent protein [GFP]) or Balb/c (H2Kd+) fetuses. For congenic IUT, B6‐GFP AFSCs were transplanted in B6 fetuses (Fig. 2A). For allogenic IUT, Balb/c AFSCs were transplanted in B6 fetuses (Fig. 2A), or B6‐GFP AFSCs were transplanted in Balb/c fetuses. Experimental protocols were approved by the Institutional Animal Care and Use Committee at The Children's Hospital of Philadelphia and University College London (PPL number: 70/7408), and followed guidelines set forth in the NIH Guide for the Care and Use of Laboratory animals, as well as the Animals (Scientific Procedures) Act (1986) and the NC3Rs Animal Research: Reporting of In Vivo Experiments guidelines 25.

### AFSC Isolation and Culture

**Figure 1:**
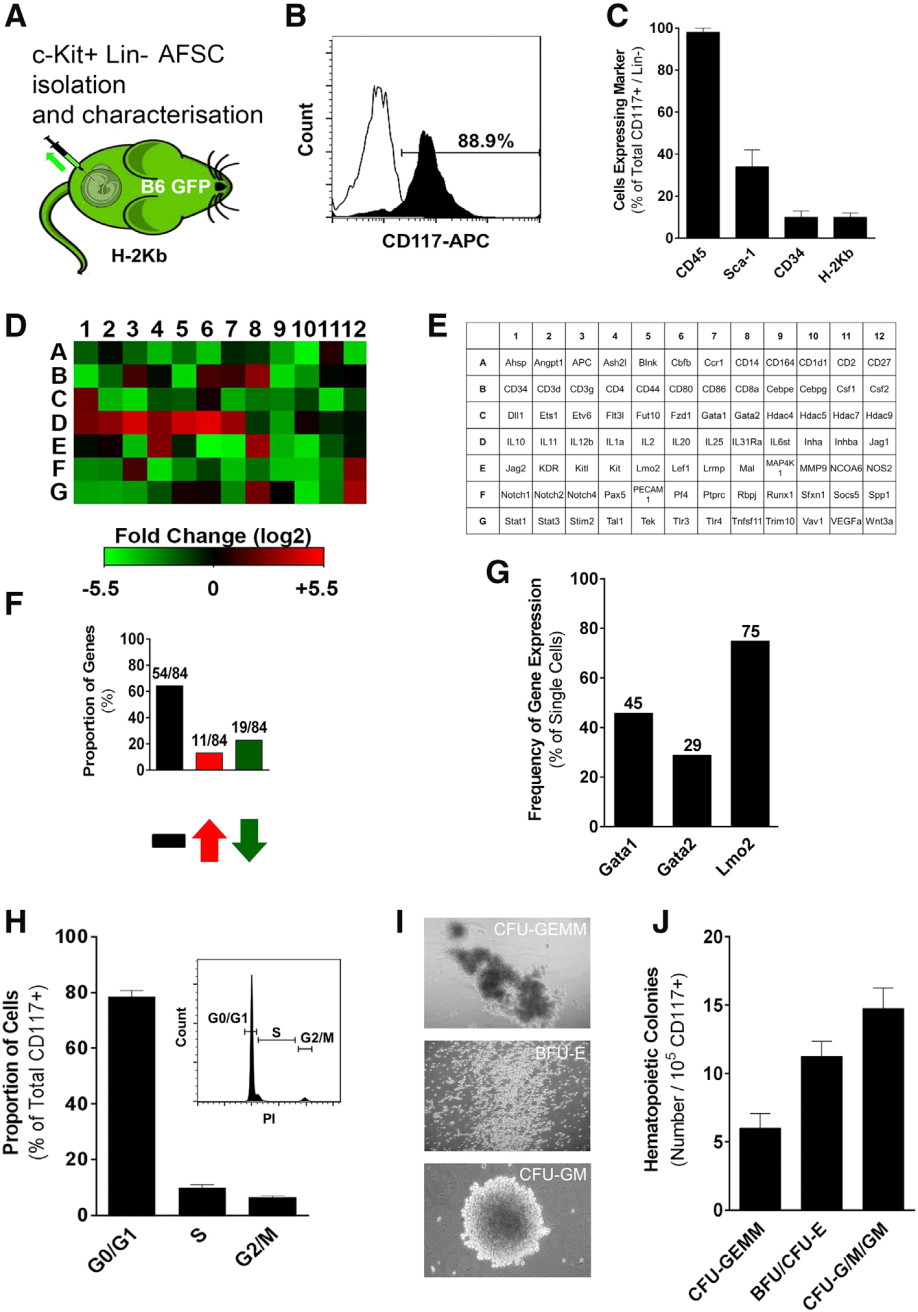
Isolation and characterization of amniotic fluid stem cells (AFSCs). **(A):** Amniotic fluid was isolated from time‐dated B6‐GFP dams at E13 with **(B)** a high level of purity based on CD117 expression (representative FACS histogram; eight independent experiments). **(C):** The phenotype of AFSC includes CD45, Sca‐1, CD34, and major histocompatibility complex class I (*n* = 8). **(D, E):** Gene array analysis of AFSC compared with adult bone marrow‐derived hematopoietic stem cells (BM‐HSCs). Red signifies upregulation, whereas green signifies downregulation of genes (representative heat map; three independent experiments). **(F):** Total number and proportion of genes that are unchanged, downregulated, or upregulated compared with adult BM‐HSCs (*n* = 3). **(G):** Percentage of AFSC that express Gata1, Gata2, and Lmo2 on single‐cell quantitative reverse‐transcription polymerase chain reaction. **(H):** Proportion of AFSC that are in the resting/quiescent (G0/G1) phase, DNA replication (S) phase, or mitotic (G2/M) phase (representative FACS histogram; six independent experiments). **(I):** Representative images (three independent experiments) of hematopoietic colonies formed by AFSC cultured in semisolid media (burst‐forming and erythroid colony‐forming units: BFU/CFU‐E [magnification: ×125]; granulocyte/macrophage colony‐forming units: CFU‐G/M/GM [magnification: ×50]; mixed granulocyte/erythrocyte/monocyte/megakaryocyte colony‐forming units: CFU‐GEMM [magnification: ×50]). **(J):** Absolute numbers of CFU‐GEMM, BFU/CFU‐E, and CFU‐G/M/GM (*n* = 3).

Amniotic fluid was collected from time‐dated B6‐GFP or Balb/c dams at E13 as described previously (see Supporting Information Methods) 26. To isolate AFSCs, cells were lineage depleted and CD117 (c‐Kit) selected by magnetic separation (magnetic‐activated cell sorting [MACS]) using commercially available mouse lineage cell depletion (antibodies against CD5, B220, CD11b, Gr‐1, 7‐4, and Ter‐119), and CD117 selection micro‐bead kits (Miltenyi Biotec, Bergisch Gladbach, North Rhine‐Westphalia, Germany; Fig. 1A).

Freshly isolated AFSCs were cultured as described previously (see Supporting Information Methods) 26. Cell growth/proliferation was quantified at 1, 3, 6, and 8 days of culture using an MTS‐based colorimetric assay (MTS Cell Proliferation Assay; Abcam, Cambridge, Massachusetts, US). After 8 days, cells were harvested and sorted for CD117 (Supporting Information Table S1) by flow cytometry (fluorescence activated cell sorting [FACS]; FACSAria, Becton Dickinson, Franklin Lakes, New Jersey, United States).

### BM Mononuclear Cell and HSCs Isolation

Low‐density BM mononuclear cells (MNCs) were separated by Ficoll gradient centrifugation. BM‐HSCs were isolated from BM‐MNCs by lineage depletion (MACS), followed by selection of CD117 and Sca‐1 double‐positive cells (CD117+, Sca‐1+, Lin−; LSK; Supporting Information Table S1) using FACS sorting (FACSAria, Becton Dickinson).

### AFSC Characterization

#### *Purity, Cell Surface Marker Expression, and Cell Cycle Analysis*

The purity of MACS‐isolated AFSCs was evaluated by FACS using a CD117 antibody (Supporting Information Table S1). Fresh and cultured CD117+ AFSCs were further characterized by assessing expression of CD45, Sca‐1, CD34, and H2Kb (Supporting Information Table S1). In order to identify the proportion of freshly isolated AFSCs that were in one of the three interphase stages of the cell cycle (G0/G1, S, G2/M), we used a propidium iodide (PI; Sigma, St. Louis, Missouri, United States)‐based protocol. Fresh AFSCs were fixed in 70% ethanol, treated with ribonuclease A, and stained with PI (400 μl of 1 mg/ml stock solution per 10^6^ cells) prior to cell cycle analysis by FACS.

#### *Hematopoietic Gene Expression Analysis*

Single cell quantitative reverse‐transcription polymerase chain reaction (qRT‐PCR) analysis of AFSCs was performed as described previously (see Supporting Information Methods) 27. We focused our analysis on the key hematopoietic regulators GATA1, GATA2, and Lmo2, as well as the pluripotency regulators Oct4, c‐Myc, Klf4, Sox2, and Nanog.

To further characterize the expression of hematopoietic genes in fresh and cultured AFSCs, we used a commercially available gene array (RT^2^ Profiler PCR Array; PAMM‐054Z; Qiagen, Hilden, Germany) according to manufacturer's instructions (see Supporting Information Methods). Expression of these genes in AFSCs was compared with that observed in BM‐HSCs. Data analysis was performed using online software (http://pcrdataanalysis.sabiosciences.com/pcr/arrayanalysis.php).

#### *Colony Forming Assay*

The in vitro hematopoietic potential of fresh and cultured AFSCs was evaluated using colony forming assays. A total of 10^4^ cells (fresh or cultured CD117+ AFSCs) were added to 2.5 ml of semisolid media (Methocult; Stem Cell Technologies Canada Inc., Vancouver, U.K.), plated in 35 mm dishes, and cultured in a humidified incubator at 37°C and 5% CO_2_. Colonies were characterized and counted after 14 days of culture.

### IUT

Intravenous IUT was performed at E14 as previously described (see Supporting Information Methods) 14, 15. Half of the surviving injected offspring in the allogenic group were nursed at birth by naive (noninjected) surrogate mothers in order to exclude transfer of maternal allo‐antibodies through breast milk 17.

### Donor Cell Tracking in the Fetus

In order to determine donor cell distribution in recipients following IUT, fetuses were harvested at E14 (4 hours post‐IUT), E17, and E19. Fetuses were dissected under a microscope and key hematopoietic tissues including the fetal liver, spleen, and thymus were exposed. Qualitative assessment of the presence of donor cells in these tissues was performed by detecting GFP+ cells with a fluorescent microscope.

### Donor Cell Hematopoietic Engraftment and Multilineage Differentiation

Chimerism was assessed in peripheral blood, BM, spleen, and thymus from E14 injected mice at 4 (postnatal day 28, P28), 12 (P84), and 24 (P168) weeks of age. MNCs were stained with an antibody against CD45 (Supporting Information Table S1). In experiments using B6‐GFP donor cells, engraftment was assessed as the percentage of CD45+ cells that were GFP+ by FACS, with chimerism being defined as more than 1% GFP+. In experiments using Balb/c donor cells, MNCs were stained with a H2Kd antibody (Supporting Information Table S1), and the percentage of CD45+ cells that were H2Kd+ was determined by FACS, with chimerism being defined as more than 1% H2Kd+. Qualitative confirmation of engraftment results obtained by FACS was obtained using immunohistochemistry (BM, spleen thymus collected at P28; see Supporting Information Methods). Multilineage differentiation of donor cells was assessed by FACS in donor‐derived MNCs in blood and BM at 24 weeks using antibodies against lymphoid (CD3, B220), myeloid (CD11b, Gr‐1), and erythroid (BM only; Ter119) markers (Supporting Information Table S1).

### Adaptive Immune Response Assessment

Spleen and lymph nodes were harvested from B6 (H2Kb+) mothers and their progeny that received allogenic (Balb/c; H2Kd+) AFSCs IUT (with or without fostering) at 4 weeks of age. The cellular adaptive immune response was assessed using the in vivo mixed lymphocyte reaction (MLR) as described previously (see Supporting Information Methods) 17. The frequency of alloreactive T cells in vivo was quantified as described by Suchin et al. 28.

The humoral adaptive immune response was assessed using serum from 4‐week‐old allogenic IUT recipient B6 mice and Balb/c splenocytes as described previously (see Supporting Information Methods for allo‐antibody assay) 17.

### Statistical Analysis

Data were compared using unpaired Student's *t* test, one‐way or two‐way analysis of variance (see Supporting Information Methods for details).

## Results

### Freshly Isolated AFSC Have Hematopoietic Potential

Mouse AFSCs (CD117+, Lin−) can be isolated at E13 with a high degree of purity (expression of CD117 postisolation: 88.7% ± 1.7%, Fig. 1B; expression of non‐CD45 hematopoietic lineage markers postisolation 0.9% ± 0.3%). Approximately 1 × 10^4^–5 × 10^4^ AFSCs could be isolated from each fetus (1% of live cells found in each amniotic sac) 27. Freshly isolated AFSCs demonstrated near‐universal expression of CD45 (96.8% ± 2.3%), but low levels of other hematopoietic markers (Sca‐1+: 31.3% ± 74%; CD34+: 9.6% ± 3.2%) and MHC (class I/H2Kb: 9.1% ± 1.7%; Fig. 1C). Hematopoietic gene array analysis of fresh AFSCs and comparison with adult BM‐derived HSCs demonstrated similar levels of expression in 64.3% (54/84) of examined genes, with significant upregulation and downregulation (>twofold) in 13.1% (11/84) and 22.6% (19/84) of examined genes, respectively (Fig. 1D–1F and Supporting Information Fig. S1A, S1C, S1E). Single‐cell qRT‐PCR analysis showed that the majority of fresh AFSCs (75%) expressed the key hematopoietic regulator Lmo2 with lower levels of expression of Gata1 (45%) and Gata2 (29%; Fig. 1G and Supporting Information Fig. S2A). We also looked into expression of pluripotency regulators, and found high levels of expression of Oct4 (76%), c‐Myc (45%) and Klf4 (55%; Supporting Information Fig. S2B). Only 10% of analyzed fresh AFSCs expressed Sox2, and none of the cells expressed Nanog (Supporting Information Fig. S2B). Most AFSCs were found to be in G0/G1 phase of the cell cycle (78.5% ± 2.2%), with only a small proportion in the S or G2/M phases (Fig. 1H). Finally, freshly isolated AFSCs exhibited significant clonogenic potential (32 ± 2 colonies per 10^5^ cells) when cultured in semisolid media, with formation of burst/erythroid‐, granulocyte/macrophage‐, and mixed‐colony‐forming units (BFU/CFU‐E, CFU‐G/M/GM, CFU‐GEMM, respectively; Fig. 1I, 1J). We have observed similar results in fresh AFSCs isolated from mouse strains other than B6‐GFP, including B6.SJL‐Ptprc^a^ Pepc^b^/BoyJ (CD45.1) 21 and Balb/c (Supporting Information Fig. S3A, S3B).

### IUT of Congenic but Not Allogenic AFSCs Results in Hematopoietic Engraftment

**Figure 2:**
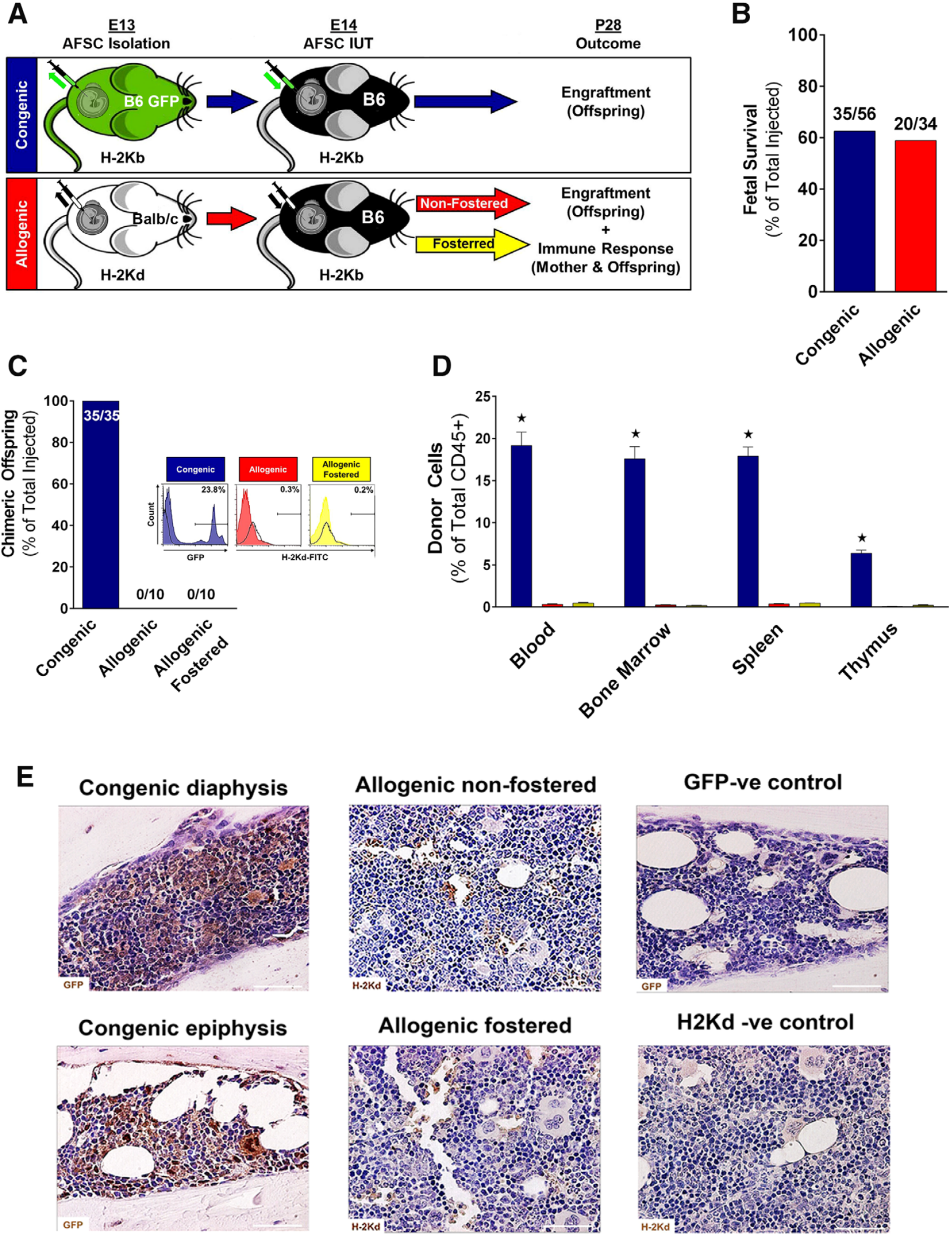
In utero transplantation (IUT) of congenic and allogenic amniotic fluid stem cells (AFSCs). **(A):** Experimental design of IUT of congenic or allogenic AFSCs into E14 fetal mice. **(B):** Fetal survival to birth after congenic and allogenic IUT injections. **(C):** Proportion of surviving animals that showed macrochimerism at P28 after IUT into congenic, allogenic‐nonfostered, or allogenic‐fostered animals (representative histograms; *n* = 35 for congenic, and *n* = 10 for each of allogenic and allogenic‐fostered). **(D):** Donor cell chimerism (expressed as percentage of total CD45+ cells) in the blood, bone marrow, spleen, and thymus at P28 after IUT into congenic (*n* = 35), allogenic‐nonfostered (*n* = 10), or allogenic‐fostered (*n* = 10) animals. **(E):** Histology of diaphysis and epiphysis in congenic and allogenic animals as well as uninjected control (representative images [magnification: ×40; scale bar: 100 μm]; six independent experiments in each group). Blue bars: congenic; red bars: allogenic nonfostered; yellow bars: allogenic‐fostered; *, *p* < .001 congenic versus allogenic and allogenic‐fostered.

In our first study, we sought to compare the potential of congenic (B6‐GFP; H2Kb+) and allogenic (Balb/c; H2Kd+) AFSCs to engraft the hematopoietic system after IUT. 10^4^ AFSCs were transplanted in E14 B6 (H2Kb+) fetuses (Fig. 2A). Fetal survival post‐IUT was 62.5% in the congenic group similar to that observed in fetuses receiving allogenic IUT (58.9%; Fig. 2B). IUT of congenic AFSCs resulted in hematopoietic macrochimerism at 4 weeks of age (P28) in 100% of surviving animals (Fig. 2C), with levels of engraftment in blood of 19.2% ± 1.6% (Fig. 2D). Similar results were observed in BM (17.6% ± 1.4%) and spleen (17.9% ± 1.1%), with lower levels of engraftment seen in the thymus (6.4% ± 0.4%; Fig. 2D). In contrast, minimal engraftment (blood: 0.3% ± 0.07%; Fig. 2C, 2D) was observed in hematopoietic tissues of animals that received allogenic AFSCs, with no animals exhibiting macrochimerism regardless of fostering. FACS results were confirmed qualitatively with immunohistochemical analysis of BM (Fig. 2E), spleen, and thymus (data not shown).

### IUT of Allogenic AFSC Results in an Adaptive Immune Response and Donor Cell Rejection

**Figure 3:**
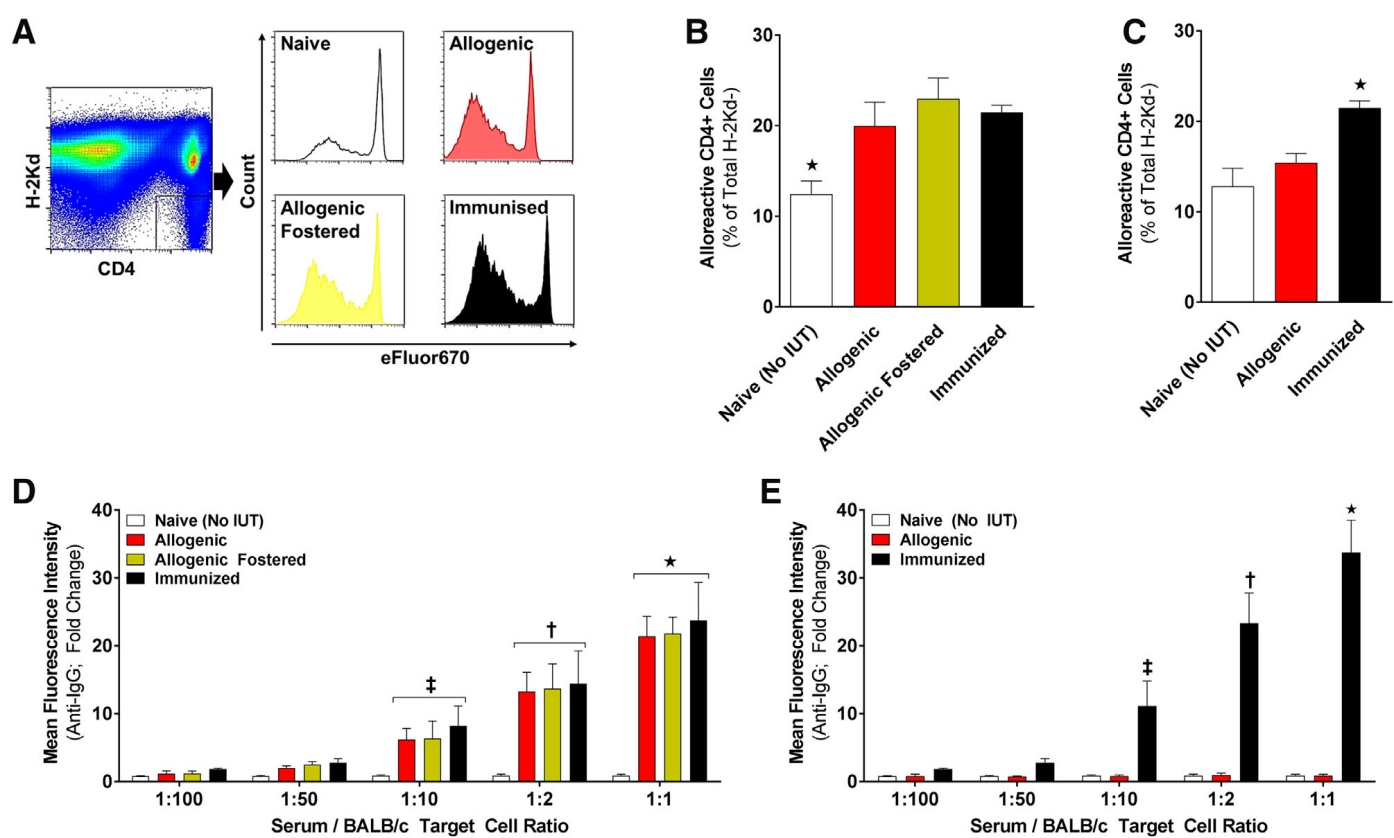
Elucidating the immune response following in utero transplantation (IUT) of allogenic amniotic fluid stem cells (AFSCs). **(A):** F1 mice (derived from mating of B6 and Balb/c mice) were euthanized 72 hours after adoptive transfer and CD4+ H2Kd− and lymphocytes were analyzed by FACS (representative gating strategy; *n* = 6 per group). **(B):** Percentage of H2Kd− cells that are alloreactive CD4+ cells in F1 mice injected with lymphocytes of naïve, allogenic, allogenic‐fostered, or immunized mice (*n* = 6). **(C):** Percentage of H2Kd− cells that are alloreactive CD4+ cells in F1 mice injected with lymphocytes from naïve or immunized mice as well as the mothers of allogenic AFSC recipients (*n* = 4 per group). **(D, E):** Mean anti‐IgG immunofluorescence intensity of mouse serum when exposed to target cells at specific ratios (*n* = 6 per group). (D): Mouse serum was used from naïve, allogenic, allogenic‐fostered, or immunized mice. (E): Mouse serum was used from naïve or immunized mice as well as the mothers of allogenic AFSC recipients (*n* = 4 per group). White bars: naive (no IUT)/negative control; red bars: allogenic nonfostered/mothers of allogenic recipients; yellow bars: allogenic‐fostered; black bars: immunized/positive control; (B): *, *p* < .001 naïve versus allogenic, allogenic‐fostered, and immunized; (C): *, *p* < .001 naive versus allogenic and immunized; (D): *, *p* < .0001; ^†^, *p* < .001; and ^‡^, *p* < .01 allogenic, allogenic‐fostered, and immunized versus naive; (E): *, *p* < .0001; ^†^, *p* < .001; and ^‡^, *p* < .01 immunized versus naive and allogenic.

To elucidate a possible role of an effector T‐cell response in the lack of chimerism after IUT of allogenic AFSCs, we performed in vivo MLR 28. The relative frequency of alloreactive T cells at 72 hours was 19.9% ± 1.0% for allogenic and 22.9% ± 0.9% for allogenic‐fostered animals (Fig. 3A, 3B). These values were significantly higher than the ones observed in naïve B6 mice (mice that did not receive IUT; 12.4% ± 0.5%; *p* < .001 versus allogenic and allogenic‐fostered), but similar to those seen in B6 mice immunized with Balb/c cells (21.5% ± 0.4%; Fig. 3B). Similar analysis in mothers of allogenic AFSCs recipients demonstrated lack of sensitization of the maternal immune system (Fig. 3C).

Serum from allogenic and allogenic‐fostered pups demonstrated significantly higher median anti‐IgG fluorescence intensity compared with negative controls (naïve) at serum‐to‐target cell ratios of 1:10, 1:2, and 1:1 (similar to that observed in immunized animals; Fig. 3D). This is consistent with antidonor allo‐antibody formation in pups that received IUT of allogenic AFSCs regardless of fostering. As was the case for alloreactive T cells, there was no evidence of a maternal humoral response to IUT of allogenic AFSCs (Fig. 3E).

### Lack of Thymic Homing Explains Rejection of Allogenic AFSC Following IUT

**Figure 4:**
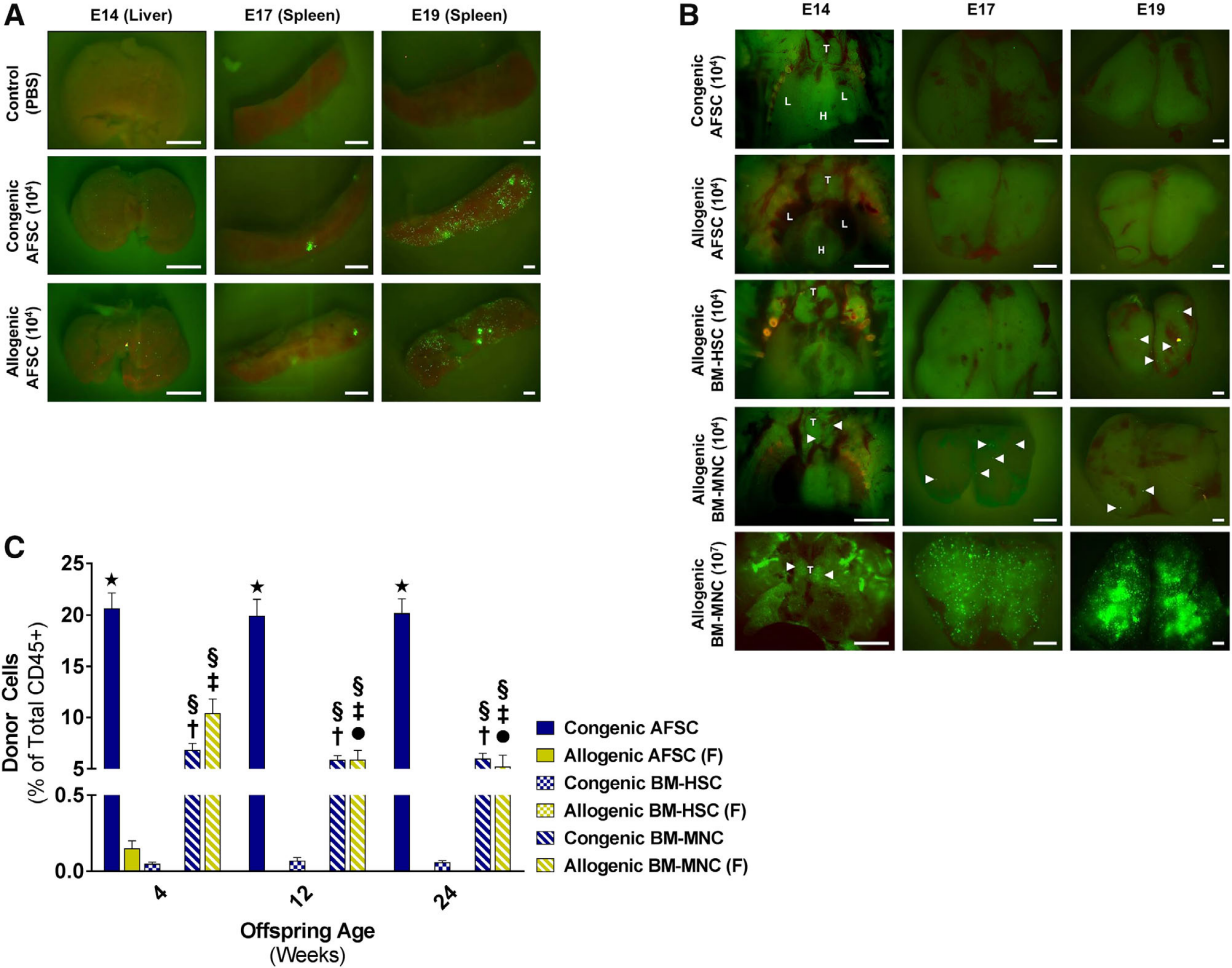
Lack of thymic homing explains the rejection of allogenic amniotic fluid stem cells (AFSCs) following in utero transplantation (IUT). **(A):** Donor cell tracking experiments after IUT of 10^4^ AFSC from B6‐GFP donors at E14 into congenic (B6) and allogenic (Balb/c) recipients. Fetal livers tracked 4 hours post‐IUT and fetal spleens tracked at E17 and E19 showed no noticeable difference in homing of AFSCs after congenic and allogenic IUT (representative images [scale bar: 200 μm]; six independent experiments). **(B):** Thymus tracking at E14 (4 hours post‐IUT), E17, and E19 after IUT of 10^4^ congenic AFSC (B6‐GFP AFSC into B6 recipients), 10^4^ allogenic AFSC (B6‐GFP AFSC into Balb/c recipients), 10^4^ allogenic bone marrow‐derived hematopoietic stem cells (BM‐HSCs; B6‐GFP BM‐HSCs into Balb/c recipients), 10^4^ allogenic BM‐mononuclear cells (MNCs), and 10^7^ allogenic BM‐MNCs (B6‐GFP BM‐MNCs into Balb/c recipients). Neither congenic nor allogenic AFSC home to the thymus at any time point during gestation (T, thymus; H, heart; L, lung; representative images [scale bar: 200 μm]; six independent experiments). **(C):** Engraftment levels (4–24 weeks of recipient age) after IUT of congenic 10^4^ AFSC (B6‐GFP AFSC into B6 recipients), 10^4^ allogenic AFSC (B6‐GFP AFSC into Balb/c recipients), 10^4^ congenic BM‐HSCs (B6‐GFP BM‐HSCs into B6 recipients), 10^4^ allogenic BM‐HSCs (B6‐GFP BM‐HSCs into Balb/c recipients), 10^7^ congenic BM‐MNCs (B6‐GFP BM‐MNCs into B6 recipients), and 10^7^ allogenic BM‐MNCs (B6‐GFP BM‐MNCs into Balb/c recipients; *n* = 6 per group). Both congenic and allogenic BM‐MNCs resulted in macrochimerism with no significant difference in long‐term engraftment between the two groups. IUT of congenic and nonallogenic AFSC resulted in macrochimerism, much higher than achieved by IUT of allogenic or congenic BM‐MNCs. In the case of BM‐HSCs IUT of congenic cells resulted in microchimerism only, with no engraftment seen when allogenic cells were used for transplantation. All animals that received IUT of allogenic cells were fostered (F) after birth. Solid blue bars: congenic AFSC; solid yellow bars: allogenic AFSC; checkered blue bars: congenic BM‐HSCs; checkered yellow bars: allogenic BM‐HSCs; striped blue bars: congenic BM‐MNCs; striped yellow bars: allogenic BM‐MNCs; *, *p* < .0001 congenic AFSC versus allogenic AFSC, congenic and allogenic BM‐HSCs (all time points); ^§^, *p* < .001 congenic and allogenic BM‐MNCs versus congenic AFSC (all time points); ^†^, *p* < .001 congenic BM‐MNCs versus allogenic AFSC, congenic and allogenic BM‐HSCs (all time points); ^‡^, *p* < .001 allogenic BM‐MNCs versus allogenic AFSC, congenic and allogenic BM‐HSCs (all time points); ^●^, *p* < .01 allogenic BM‐MNCs at 12 and 24 weeks versus allogenic BM‐MNCs at 4 weeks.

In utero tracking experiments were used to investigate the rejection and lack of engraftment of allogenic AFSCs. In order to be able to detect allogenic AFSCs easily in fetal tissues, we performed IUT of B6‐GFP cells in Balb/c donors. Donor cell tracking was performed at: (a) E14 (4 hours post‐IUT) to assess homing of AFSCs to the fetal liver and thymus, (b) E17 to evaluate migration of cells to the fetal spleen, and determine whether donor cells could be found in the thymus prior to closure of the “thymic window” for development of central (deletional) tolerance, and (c) E19 to assess donor cell distribution immediately before birth (Fig. 4A, 4B). Both congenic and allogenic AFSCs (10^4^ per fetus) homed to the fetal liver soon after IUT, proliferated, and migrated to the fetal spleen with no obvious differences detected between groups at any analysis time point (Fig. 4A). Following this, we focused our attention on the thymus and compared homing of AFSCs to that of allogenic BM‐MNCs (10^7^ B6‐GFP BM‐MNCs per Balb/c fetus that result in stable hematopoietic engraftment across immune barriers post‐IUT 17, or 10^4^ per fetus to control for cell number), and that of allogenic BM‐HSCs (CD117+, Sca‐1+, Lin−; 10^4^ B6‐GFP BM‐HSCs per Balb/c fetus as stem cell control group). A significant number of donor (GFP+) cells were detected in the thymus post‐IUT of either 10^4^ or 10^7^ BM‐MNCs from as early as 4 hours following transplantation (Fig. 4B). This was in contrast to what we observed for both congenic and allogenic AFSCs, which, despite engrafting in other hematopoietic organs, did not home to the thymus at any time point (Fig. 4B). Surprisingly, when BM‐HSCs were used for IUT, donor cells were not seen in the thymus until E19 (Fig. 4B), which raised questions about the ability of such an enriched donor cell population to induce tolerance in this setting.

To address this issue, we assessed engraftment at 4, 12, and 24 weeks of offspring age in all experimental groups included in our tracking analysis. As we have reported previously 14, IUT of congenic and allogenic BM‐MNCs resulted in macrochimerism in blood with no significant differences detected between the two groups in any of the analysis time points (Fig. 4C). In contrast, IUT of congenic but not allogenic AFSCs resulted in stable, long‐term hematopoietic macrochimerism in 100% of transplanted animals (Fig. 4C), with significantly higher levels of engraftment achieved in congenic AFSCs animals compared with recipients of congenic or allogenic BM‐MNCs (*p* < .001; Fig. 4C). The lack of engraftment of B6‐GFP AFSCs following IUT in Balb/c fetuses is similar to what we observed when Balb/c AFSCs were transplanted in B6 fetuses (Fig. 2C, 2D). No engraftment was observed when enriched allogenic BM‐HSCs were used, with only microchimerism achieving post‐IUT of congenic BM‐HSCs (Fig. 4C). As was the case with allogenic AFSCs (Fig. 3), there was evidence of an adaptive immune response in allogenic BM‐HSCs recipients resulting in rejection of donor cells (Supporting Information Fig. S4A, S4B). These findings confirm the requirement for timely (prior to E17) donor cell homing to the thymus for induction of central/deletional tolerance and explain the paradoxical rejection and lack of engraftment of allogenic AFSCs.

### AFSC Can Be Expanded Without Losing Their Hematopoietic Potential

**Figure 5:**
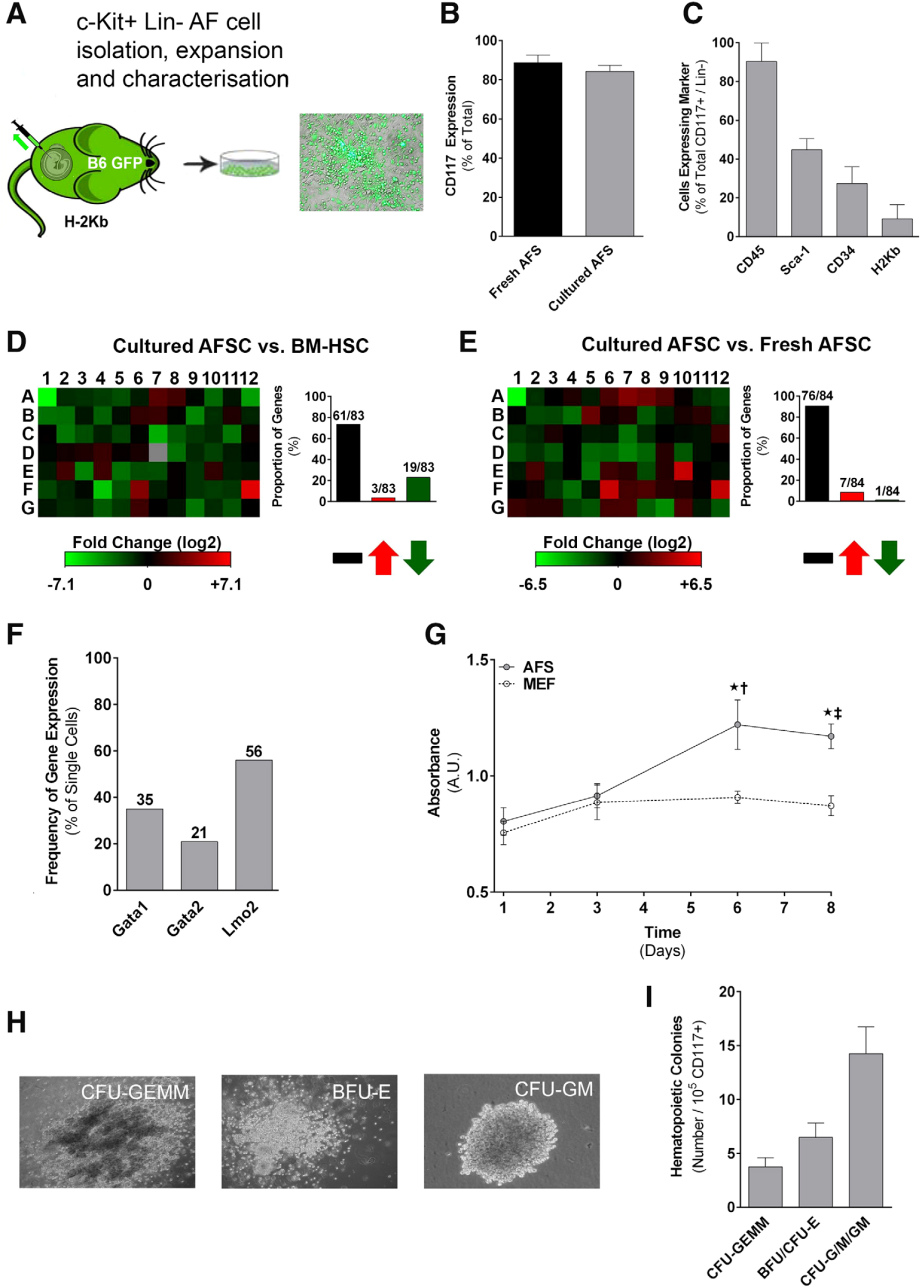
In vitro expansion of amniotic fluid stem cells (AFSCs) without loss of their hematopoietic potential. **(A):** Culture of c‐Kit+/Lin− AF cells on mitotically inactivated MEF (representative image; >50 independent experiments). **(B):** CD117 expression does not change after 8 days of culture and expansion (*n* = 10 in each group). **(C):** Expression of cell surface markers such as CD45, Sca‐1, CD34, and H2Kb (*n* = 10). **(D):** Gene array assay of cultured AFSC in comparison with fresh AFSC (representative heat map; three independent experiments). **(E):** Gene array assay of cultured AFSC in comparison with bone marrow‐derived hematopoietic stem cell (representative heat map; three independent experiments). **(F):** Single cell quantitative reverse‐transcription polymerase chain reaction analysis of cultured AFSC showing expression of the hematopoietic regulators Lmo2, Gata1, and Gata2. **(G):** MTS assay of AFSC and MEF (*n* = 6). **(H, I):** Formation of mixed colony‐forming units (CFU)‐granulocyte/erythrocyte/monocyte/megakaryocyte (GEMM; magnification: ×50), burst‐forming units (BFU)/erythroid CFU (magnification: ×125), and granulocyte/macrophage colony‐forming units (CFU‐G/M/GM; magnification ×50) from cultured AFSC, demonstrating retained multipotent hematopoietic potential ([H]: representative images; [I]: graph summarizing data from three independent experiments). (G): *, *p* < .001 AFSC days 6 and 8 versus day 1; ^†^, *p* < .01 AFSC day 6 versus day 3; ^‡^, *p* < .05 AFSC day 8 versus day 3.

Having established the remarkable hematopoietic capacity of AFSCs post‐IUT, we tested their in vitro expansion potential. CD117+/Lin− AFSCs were successfully cultured on a feeder layer of mitotically inactivated MEF (Fig. 5A) 26. The present culture protocol allows expansion without loss of CD117 expression following 8 days of culture (CD117+; cultured AFSCs: 84.3% ± 0.9%; Fig. 5B). Further characterization showed that CD45 expression was also maintained (90.4% ± 9.5%; Fig. 5C), with levels of expression of other hematopoietic markers raised compared with freshly isolated AFSCs, and low levels of MHC class I expression (Fig. 5C). Almost all cells remained negative for non‐CD45 hematopoietic lineage markers (Lin−: 0.6% ± 0.2%). Hematopoietic gene array analysis of cultured AFSCs and comparison with BM‐HSCs showed similar levels of expression in 73.5% (61/83) of examined genes, with significant upregulation in only 1.2% (1/83) and downregulation in 22.9% (19/83) of examined genes (Fig. 5E and Supporting Information Fig. S1B, S1D, S1F). Direct comparison of hematopoietic gene expression in fresh and cultured AFSCs showed similar levels of expression in 90.5% (74/84) of examined genes (Fig. 5D). Single cell qRT‐PCR demonstrated expression of the hematopoietic regulators Lmo2 (56%), Gata1 (35%), and Gata2 (21%; Fig. 5F and Supporting Information Fig. S2A). We also looked into expression of pluripotency regulators and found that although c‐Myc and Klf4 expression was maintained in cultured AFSCs compared with fresh (65% and 58%, respectively), Oct4 expression was significantly reduced (13%; Supporting Information Fig. S2C). Similar to the fresh AFSCs, only 4% of analyzed cultured AFSCs expressed Sox2, and none of the cells expressed Nanog (Supporting Information Fig. S2C). Microscopic analysis of cultured AFSCs showed minimal proliferation for the first 3 days, followed by significant growth thereafter. To assess cell growth formally, CD117+ cells were isolated and counted at day 8 of culture, demonstrating a fourfold rise in the number of cells over the 8‐day culture period (fold‐increase: 4.1 ± 0.3). These findings were confirmed with MTS‐based colorimetric analysis (Fig. 5G), and are consistent with AFSCs expansion. Finally, cultured AFSCs demonstrated in vitro hematopoietic function in semisolid media (Fig. 5H, 5I) similar to that of fresh AFSCs. Using this culture system, we have managed to expand murine AFSCs for up to five passages (cell passage when 80% confluence achieved, typically after 6–8 days of culture) without any effect on cell surface phenotype or in vitro hematopoietic potential.

### IUT of Cultured AFSC Results in Long‐Term, Multilineage Hematopoietic Engraftment

**Figure 6:**
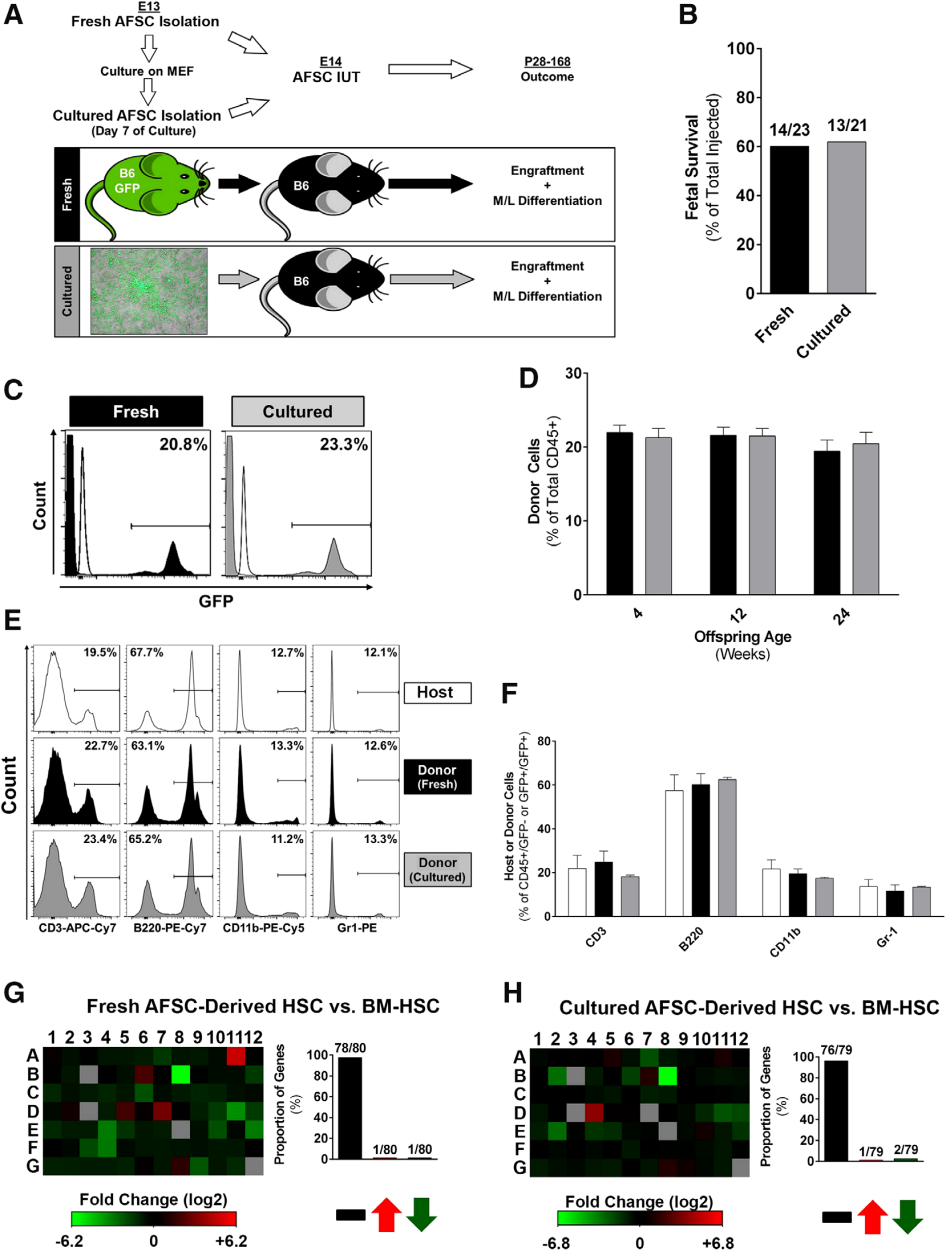
In utero transplantation (IUT) of cultured amniotic fluid stem cells (AFSCs) results in long‐term multilineage hematopoietic engraftment. **(A):** Experimental design for IUT of fresh and cultured AFSC (B6‐GFP; CD117+, Lin−, H2Kb+; 10^4^ per fetus), performed at E14. Chimerism was checked up to 24 weeks post‐IUT. **(B):** Fetal survival to birth after IUT of fresh versus cultured AFSC. **(C):** Four‐week chimerism analysis showed 100% engraftment in both groups (representative FACS histograms; fresh AFSC: 14/14; cultured AFSC: 13/13) with similar levels of macrochimerism (representative FACS histograms). **(D):** Engraftment levels between fresh (*n* = 14) versus cultured AFSC (*n* = 13) at 4, 12, 24 weeks post‐IUT. **(E, F):** Multilineage reconstitution of transplanted AFSC (fresh and cultured) compared with donor lineages (representative FACS histograms; *n* = 8 per group). **(G, H):** Hematopoietic gene array analysis comparing donor‐derived (fresh or cultured) AFSC and host bone marrow‐derived hematopoietic stem cells (representative heat maps; three independent experiments). Black bars: fresh AFSC/fresh AFSC‐derived; gray bars: cultured AFSC/cultured AFSC‐derived; white bars: host‐derived.

Our final in vivo study determined the ability of fresh and cultured AFSCs to engraft the hematopoietic system long‐term post‐IUT. We compared hematopoietic chimerism achieved with fresh and cultured AFSCs for up to 24 weeks post‐IUT (Fig. 6A). Fetal survival was 61.9% in the cultured AFSCs group, similar to fresh AFSCs recipients (Fig. 6B). Hematopoietic macrochimerism was achieved in 100% of animals that received fresh or cultured AFSCs, with similar levels of engraftment in blood observed in the two experimental groups at 4 weeks of age (P28; fresh AFSCs: 21.9% ± 1.0%; cultured AFSCs: 21.3% ± 1.2%; Fig. 6C, 6D). Hematopoietic engraftment was stable in fresh and cultured AFSCs recipients at 12 (P84) and 24 weeks of age (P168) with no differences observed between groups (Fig. 6D). Moreover, multilineage analysis of donor cells in recipient blood at 24 weeks of age (P168; Fig. 6E) demonstrated normal differentiation to lymphoid (CD3+, B220+) and myeloid (CD11b+, Gr‐1+) lineages similar to that observed in the host (Fig. 6E, 6F).

Our findings in blood were confirmed in BM and spleen at 6 months; there were no differences in BM engraftment between groups (fresh AFSCs: 18.7% ± 1.3%; cultured AFSCs: 17.9% ± 1.7%), and donor cells demonstrated differentiation to all hematopoietic lineages including erythroid (Ter119+; Supporting Information Fig. S5A–S5C). We also studied the HSCs “compartment” of BM‐MNCs, and isolated donor‐derived (GFP+) and host (GFP−) BM‐HSCs (CD117+, Sca‐1+, Lin−; LSK; see Supporting Information Fig. S6 for FACS gating strategy). The proportion of BM‐HSCs within donor‐derived (GFP+) and host (GFP−) BM‐MNCs was balanced/similar (proportion of fresh AFSCs‐derived BM‐HSCs within GFP+ MNCs: 0.11% ± 0.06%, Supporting Information Fig. S6A; proportion of cultured AFSCs‐derived BM‐HSCs within GFP+ MNCs: 0.09% ± 0.03%, Supporting Information Fig. S6B; proportion of host BM‐HSCs within GFP− MNCs: 0.10% ± 0.09%, Supporting Information Fig. S6A, S6B), and hematopoietic gene array analysis showed similar levels of expression in almost all of the examined genes when donor‐derived and host BM‐HSCs were compared (Fig. 6G, 6H).

## Discussion

We demonstrate here that IUT of autologous cultured AFSCs resulted in long‐term, multilineage hematopoietic engraftment. On the contrary, IUT of allogenic AFSCs led to an adaptive immune response and rejection most likely related to the lack of thymic homing of donor cells during gestation.

C‐Kit+/Lin− AFSCs isolated from mouse amniotic fluid showed hematopoietic characteristics with expression of hematopoietic markers and key hematopoietic regulators. Most AFSCs were in quiescence, in keeping with a primitive HSCs phenotype, which is also evident when their genetic profile is compared with more mature BM‐HSCs. AFSCs exhibited significant clonogenic potential, producing colonies from all three hematopoietic lineages when cultured in semisolid media. This is in keeping with what we have previously demonstrated 19, 21, and further highlights the potential of obtaining fetal HSCs from an easily accessible source during gestation 20. Most importantly, we proved here that following 8 days of culture and a fourfold rise in the number of CD117+ cells, AFSCs maintained a similar phenotype and exhibited significant hematopoietic potential equal to that of freshly isolated AFSCs (similar numbers of hematopoietic colonies were observed when identical numbers of fresh and cultured AFSCs were placed in semisolid media).

Remarkably, both freshly isolated and expanded congenic AFSCs showed high engraftment potential in vivo. In particular, 100% of animals receiving intravenous IUT of congenic 10^4^ fresh or cultured AFSCs showed stable hematopoietic macrochimerism for up to 6 months, with engraftment in blood and BM exceeding 20%. This remarkable engraftment potential may also be related to the intravenous injection technique which is superior to previously described intraperitoneal delivery and is more applicable to possible clinical translation 13, 14, 15. This is also demonstrated by our recently published preliminary study in which intraperitoneal IUT of 10^4^ congenic AFSCs per fetus resulted in long‐term hematopoietic engraftment only of around 10%, which is approximately half to that achieved in the present study using intravenous IUT 21. Multilineage long‐term engraftment equivalent to that observed in the host was confirmed in the recipient groups. Indeed, given the relatively small number of AFSCs in the amniotic fluid, clinical application in the setting of IUT for the treatment of hematological disorders will require adequate expansion without loss of their hematopoietic characteristics. This finding opens‐up the possibility of using AFSCs for the treatment of congenital hematological or other inherited disorders 18, 19, 23, 26. In particular, it would be advantageous to combine cell and gene therapy with the subsequent correction and expansion of AFSCs, which can be given back to the same fetus with the aim of correcting hematological or other monogenic diseases before birth 22. Clinically applicable ultrasound guided IUT of transduced autologous AFSCs has already been achieved in fetal sheep with a low complication rate 22, 29. Both cord blood and BM‐derived HSCs have shown the ability for limited expansion maintaining their hematopoietic potential 30, 31, 32, 33. However, the methods described did not work with AFSCs expansion, leading to inefficient replication and loss of their potential. In keeping in with their more primitive phenotype, we adopted here a method described previously to expand pluripotent stem cells, and we have observed a remarkable proliferative potential of the AFSCs while maintaining both in vitro and in vivo hematopoietic potential.

Having established the high engraftment potential of AFSCs, we sought to explore further the possibility of using allogenic AFSCs. This approach could be advantageous for congenital diseases because (in combination with in vitro expansion) it would allow banking of clinical‐grade, cost‐efficient cell lines, and would preclude the need for genetic correction 20. However, we confirmed with this study that intravenous IUT leads to minimal engraftment of allogeneic AFSCs in hematopoietic tissues (similar to intraperitoneal IUT; 21). These unexpected results were in contrast to what we and others reported for allogeneic BM‐MNCs 17 as well as fetal liver‐derived MNCs 34 injected in utero, and led us to investigate different mechanisms which could have resulted in the rejection of allogenic AFSCs post‐IUT. When considered together, our alloreactive T‐cell and allo‐antibody data confirm our recently published preliminary findings 21, and are consistent with an adaptive immune response in allogenic AFSCs recipient pups resulting in rejection of donor cells. Although separate third‐party antigen control experiments were not performed, the results obtained from naïve (negative control) and immunized (positive control) groups in MLR and allo‐antibody experiments confirm direct immunization as the cause of allogenic AFSCs rejection. Moreover, fostering of newborns in the allogenic group with noninjected surrogate mothers to negate any maternal immune response had no effect on chimerism, which is also in contrast to what has been reported previously using allogenic BM‐MNCs 17. Further studies confirmed that there was no evidence of a maternal cellular or humoral response to IUT of allogenic AFSCs. The absence of sensitization of the maternal immune system and lack of effect of fostering on engraftment suggest that our findings are due to the failure of induction of central/deletional tolerance in the offspring and not due to maternal factors 17, although we did not investigate placental trafficking of maternal cells that has been previously shown to result in loss of hematopoietic chimerism post‐IUT 34. These results were somewhat paradoxical and might be related to the more immature phenotype of ASFC, or the comparatively small cell number used for IUT. Finally, the present experimental series provides further mechanistic insight for the rejection of donor AFSCs following allogenic IUT. We here demonstrate that, both for AFSCs and BM‐HSCs, there is a requirement for timely (likely prior to E17; BM‐HSCs and/or their progeny home to the thymus but not until E19) donor cell homing to the thymus for induction of central/deletional tolerance 4, 5, 6. The lack of timely migration of the AFSCs or their progeny to the thymus may explain their rejection and lack of engraftment.

One of the limitations of our study relates to the strains of mice used in the present experimental series. Allogenic IUT was performed using Balb/c AFSCs transplanted into B6 recipients and congenic IUT utilized B6‐GFP cells administered in B6 mice. Thus, some of our findings could simply be due to strain differences in the AFSCs themselves or to decreased sensitivity of the detection method in the allogeneic strain combination. Although we did not perform a formal comparison of Balb/c and B6‐GFP‐derived AFSCs, we have characterized freshly isolated AFSCs from a variety of mouse strains (including Balb/c; Supporting Information Fig. S3) and shown similar cell surface phenotype as well as in vitro hematopoietic potential 21. Moreover, lack of engraftment of allogenic AFSCs was also observed when B6‐GFP were transplanted in Balb/c fetal recipients (Fig. 4), suggesting that our findings are due to rejection of allogenic donor cells and not due to strain‐related differences and/or methodological issues. In addition, the lack of tolerance induction post‐IUT of allogenic AFSCs could be due to the inability (rather than delay) of these cells to generate progeny able to present antigen to the thymus. However, this is unlikely based on the in vitro and in vivo multilineage hematopoietic potential of these cells, as well as the adaptive immune response‐mediated rejection of allogenic BM‐HSCs (which, by default, have the ability to generate antigen‐presenting cells). It is possible that a specific “tolerance‐inducing cell” must be cotransplanted with stem cells (AFSCs, BM‐HSCs, or otherwise) in order for allogenic IUT to be successful; in vitro generation of such cells is likely to require special (differentiation‐inducing) culture conditions, as AFSCs‐derived antigen presenting cells do not seem to be a by‐product of our culture system (although we did not look formally, the vast majority of expanded cells are cKit+ and hematopoietic lineage negative). The latter, in addition to the time‐course of rejection of AFSCs following allogenic IUT, is under investigation in our laboratory. Finally, the findings of the present experimental series may be limited to the specific subpopulation of amniotic fluid‐derived stem cells. Although prenatal therapies targeting diseases of hematopoiesis would utilize cKit+/Lin− AFSCs, cKit− amniotic fluid‐derived mesenchymal stem cells (AFMSC) could be utilized for in utero treatment of inherited disorders such as osteogenesis imperfecta 20, 23, 35. Allogenic IUT may be feasible in the latter setting, as the well‐recognized immunomodulatory properties of MSC (including AFMSC; 36) may facilitate successful engraftment across immune barriers 20, 23, 35.

Clinical application of AFSCs in the setting of IUT would necessitate demonstration that human AFSCs can be expanded in the laboratory without losing their hematopoietic potential, and have similar characteristics following prenatal transplantation. Amniocentesis clinical samples obtainable from 15 weeks of gestation contain approximately 1% AFSCs (1,000–2,000 CD117+ AFSCs in 2 ml amniotic fluid). Extrapolating from murine IUT results, we estimate that at least 12 population doublings will be required for sufficient IUT at 20 weeks of gestation (fetal weight 300 g, 40,000 AFSCs/g). Using congenic AFSCs allows IUT at a later time point (20 weeks gestation versus 14 weeks when allogenic cells are used; the latter is equivalent to E17 in mice), which is both clinically desirable (due to the potential complications of IUT during early gestation) and provides a 5‐week period for AFSCs expansion and gene engineering. Identification of the optimal human AFSCs expansion conditions while maintaining desirable cellular characteristics is currently a focus of investigation in our laboratory. Gene engineering of autologous AFSCs may have immunological implications following IUT, as it will ultimately lead to introduction of “foreign” protein (e.g., α or β globins) after successful engraftment of autologous cells in hematopoietic niches and subsequent maturation/lineage commitment. The latter may have significant implications on efficacy of the proposed therapeutic approach. In the setting of congenic/autologous‐like murine IUT, the presence of “foreign” protein (required to detect donor cells in immunologically matched congenic hosts; e.g., GFP in the current series of experiments, or CD45.1 in previously published work from out laboratory 21) did not affect engraftment or multilineage differentiation of donor cells with no evidence of an adaptive immune response. Despite these reassuring findings and given the limitations of murine IUT models, we and others are utilizing animal models of prenatal transplantation that resemble human IUT more closely in order to address this and other important issues prior to clinical application.

## Conclusion

Our findings suggest that both freshly isolated and expanded AFSCs could be used as an autologous stem cell source for the prenatal treatment of congenital hematological disorders. This would require sampling of amniotic fluid with gene correction and subsequent expansion of AFSCs before selection and IUT via intravenous injection at the optimal gestational age for engraftment and antigen presentation to the fetal thymus. In fact, our study demonstrates far greater hematopoietic potential in the fetal environment for a given cell dose of AFSCs compared with adult BM‐derived MNCs, as well as enriched BM‐HSCs, although formal limiting dilution studies were not performed. However, in contrast to congenic (autologous‐like) AFSCs, allogenic AFSCs did not engraft because of lack of induction of tolerance in the fetus. The latter is related to the inability of AFSCs or their progeny to migrate to the thymus for timely presentation of donor antigen. These findings reveal the remarkable hematopoietic potential of AFSCs in the setting of autologous/congenic IUT, and confirm the requirement for induction of central tolerance for allogenic IUT to be successful.

## Author Contributions

S.P.L.: conception and design, financial support, provision of study material, collection of data, data analysis/interpretation, manuscript writing, final approval of manuscript; P.S.: conception and design, collection of data, data analysis/interpretation, manuscript writing, final approval of manuscript; E.B., C.F., M. Piccoli, M. Pozzobon: provision of study material, data analysis/interpretation, final approval of manuscript; S.S., A.T., A.G.K., J.D.S., N.J.A.: collection of data, final approval of manuscript; H.L., C.G.F., A.I.B.S.D.: collection of data, data analysis/interpretation, manuscript writing, final approval of manuscript; A.J.T., P.B., W.H.P.: data analysis/interpretation, final approval of manuscript; A.L.D.: conception and design, financial support, data analysis/interpretation, final approval of manuscript; A.W.F., P.D.C.: conception and design, financial support, provision of study material, data analysis/interpretation, manuscript writing, final approval of manuscript.

## Disclosure of Potential Conflicts of Interest

A.J.T. declared advisory role and stock ownership interest with Orchard Therapeutics, Rocket Pharma, Generation Bio—no relation to current project. A.L.D. declared employment at University College London as professor of Obstetrics and Maternal Fetal Medicine, Scientific Trustee of Tommy's Charity, consultant for Micrgen providing advice about orphan disease designation, honoraria from Hologic, Inc. and a member of the Hologic Perinatal Advisory Board, and recipient of grants from Wellcome Trust, EPSRC, MRC, Action Medical Research, and other charities. The other authors indicated no potential conflicts of interest.

## Supporting information


**Appendix S1**: Supporting information

## Data Availability

The data that support the findings of this study are available from the corresponding author upon reasonable request.
