# Supplementary material for: In Utero Transplantation of Expanded Autologous Amniotic Fluid Stem Cells Results in Long‐Term Hematopoietic Engraftment
Source: Stem Cells. 2019 Jul 29;37(9):1176–88. doi: 10.1002/stem.3039 (PMC6773206; doi:10.1002/stem.3039)
Supplement: Supplementary file 1 — Appendix S1: Supporting information [file STEM-37-1176-s001.pdf]

SUPPLEMENTAL METHODS

Murine Amniotic Fluid Collection

Time-dated C57BL/6TgN(act-EGFP)OsbY01 (B6-GFP) or Balb/c dams were culled at E13, a midline laparotomy was performed, and the uterus was removed. The uterine wall was carefully dissected in a tissue culture hood and amniotic sacs were exposed. Each sac was carefully opened with dissecting scissors and the amniotic fluid was collected and filtered using a 40μm filter.

Amniotic Fluid Stem Cell Culture

10^5 amniotic fluid stem cells (AFSC) were plated onto a feeder layer of mitomycin C-treated mouse embryonic fibroblasts (MEF) in DMEM knockout (Life Technologies, USA) supplemented with 15% heat-inactivated FBS, 100mM nonessential amino acids (Life Technologies, USA), 2mM L-glutamine (Sigma, USA), 50units/ml penicillin (Sigma, USA), 50mg/ml streptomycin (Sigma, USA), 0.01mM 2-mercaptoethanol (Sigma, USA), 20ng/ml stem cell factor (SCF) (Peprotech, USA), and 20ng/ml leukemia inhibitory factor (LIF) (Sigma, USA). Cells were cultured at 37°C and 5% CO_2, and media was changed every 48 hours.

Amniotic Fluid Stem Cell Hematopoietic Gene Analysis

Single Cell Quantitative Reverse-Transcription Polymerase Chain Reaction
We focused our analysis on three key hematopoietic regulators (GATA1, GATA2, Lmo2), and their expression in AFSC was compared to that observed in BM-HSC. 28S ribosomal RNA was used as endogenous control.

Single fresh or cultured AFSC were FACS-sorted into 96-well plates and were frozen immediately at -80°C until quantitative reverse-transcription polymerase chain reaction (qRT-PCR) analysis was performed. Cells were lysed by heating to 65°C for 2 minutes followed by cooling to 4°C in a thermocycler (Eppendorf, UK). Single cell lysates were individually subjected to RT using the High Capacity cDNA Synthesis kit (Applied Biosystems, UK). 10μl volume of RT mix containing 12.2μM each of the 3' primer A for all the five genes (Table S2), 1.5μl buffer 10X (Applied Biosystems, UK), 1mM dNTPs, 0.75μl of RNAse-Out™ (Life Technologies, USA), and 0.75μl of Multiscribe Reverse Transcriptase (Applied Biosystems, UK) was added directly to 5μl of the single cell lysate (in phosphate-buffered saline; PBS) in each well and reverse transcribed (Cycling conditions: 25°C for 10min, 37°C for 120min, 85°C for 5min).

The first round of PCR (PCR 1) involved amplifying the cDNAs obtained from RT. 70μl of PCR mix containing buffer 10X, 2mM MgCl2, 2.5mM dNTPs, 1μl of AmpliTaq Gold DNA polymerase (all from Applied Biosystems, UK), and 25μM each of primers A and B for all the 4 genes (Table S2) was added directly to 15μl cDNA mix in each well, amplified

[cycling conditions: denaturation at 95°C for 10 minutes and 15 cycles of amplification (45 seconds at 95°C, 1 minute at 60°C, and 1 minute 30 seconds at 72°C)], and stored at -20 degrees.

qRT-PCR was carried out separately for each of the genes using a SYBR Hi-ROX kit (Bioline, London, UK) and 4ul of each of the single cell PCR 1 products. 20µl of qRT-PCR mix (2X SYBR green mix and 25µM each of primers A and C for the specific gene; Table S2) was added to 4µl of PCR 1 product and amplified [cycling conditions: denaturation at 95°C for 10 min, and 40 cycles of amplification (15 seconds at 95°C, 15 seconds at 60°C, and 15 seconds at 72°C). Melting curves were checked to ensure the specificity of the amplification products.

Hematopoietic Gene Array Analysis

We utilized a commercially available gene array (RT^2 Profiler PCR Array; PAMM-054Z; Qiagen, UK). This array profiles the expression of 84 genes related to the development of hematopoietic lineages from stem cells, including cytokines, signalling molecules/regulators, as well as blood cell lineage-specific molecular markers. RNA was purified from fresh and cultured AFSC as well as fresh BM-HSC using the RNeasy Micro kit (Qiagen, Manchester, UK), and RNA concentration and purity were determined by UV spectrophotometry. 50ng of RNA were used for each downstream reaction. Residual genomic DNA contamination from each sample was removed by incubating the sample with the genomic DNA elimination mix in a final volume of 10µl at 42°C for 5 minutes followed by snap cooling on ice. 10µl of the RE3 reverse transcription mix along with control RNA (RT^2 First Strand kit; Qiagen, Manchester, UK) was added to the 10µl of the genomic DNA-free RNA sample, incubated at 42°C for 15 minutes and then the reaction was stopped by incubating at 95°C for 5 minutes. 91µl of RNAse-free water was added to the 20µl of cDNA sample. To 102µl of the diluted cDNA sample, 1350µl of 2X RT2 SYBR GREEN mastermix (Qiagen, Manchester, UK) was added (qRT-PCR mix) and transferred to a array loading reservoir. 25µl of the qRT-PCR mix was dispensed into each well of the 96-well RT^2 profiler array using a multichannel pipette and mixed well. The 96-well plate was then sealed using an optical adhesive film (Applied Biosystems, Birchwood, UK), spun at 1000g for 1 minute to remove bubbles, and amplified in a Step-one Plus (Applied Biosystems, Birchwood, UK) [cycling conditions: denaturation at 95°C for 5 minutes followed by 40 cycles of amplification (95°C for 15 seconds, and 60°C for 1 minute)]. Melting curve analysis was done to verify PCR specificity.

In Utero Transplantation

A midline laparotomy was performed at E14 under isoflurane anesthesia and the uterine horns were exposed. The vitelline vein was identified with a dissecting microscope and each fetus was injected with 10^4 AFSC, 10^4 BM-HSC, or 10^7 BM-MNC from appropriate donors in 20µl of PBS. A successful intravenous injection was confirmed by visualization of clearance of the blood in the vein by the injectate and the absence of extravasation at

the site of injection. Following completion of the injections, the uterus was returned to the maternal peritoneal cavity and the abdomen closed with two layers of absorbable sutures. All recipient animals were weaned from their mothers at 4 weeks of age.

Assessment of AFSC Hematopoietic Engraftment by Immunohistochemistry

Qualitative confirmation of engraftment results obtained by FACS was obtained using immunohistochemistry; bone marrow samples from congenic and allogenic AFSC recipients (collected at P28) were used for this analysis. Formalin-fixed bones were decalcified using Immunocal (StatLab, USA) and then paraffin-embedded. Slides of 4-5µm sections were then made, de-paraffinized, and rehydrated. Heat-induced epitope retrieval was performed in a microwave in target retrieval buffer (pH 6.0; DAKO, USA) followed by a 30-minute cool-down period. Slides were then placed in TBST buffer (Thermo Fisher Scientific, USA) prior to assay. The following steps were performed at room temperature with TBST buffer rinses between steps. Non-specific protein binding was blocked with SuperBlock (Thermo Fisher Scientific, USA) for 30 minutes. Slides were incubated with primary rabbit anti-GFP antibody (Invitrogen, California, USA) 1:400 and anti-H-2Kd (BD Pharmingen, USA) overnight at 4°C. The next day slides were warmed in TBST for 10 minutes and then incubated for 30 minutes at room temperature with SuperPicture HRP Polymer conjugated anti-rabbit antibody (Invitrogen, USA). The chromogene used was 3’3’ diaminobenzidine (DAB; Vector Lab, USA). Slides were counterstained with hematoxylin (Thermo Fisher Scientific, USA), dehydrated in graded alcohol, cleared in xylene, and mounted in Permount (Thermo Fisher Scientific, USA).

T Cell Alloreactivity In Vivo (In Vivo Mixed Lymphocyte Reaction)

Spleen and lymph nodes (axillary, inguinal, cervical, para-aortic, and mesenteric) were harvested from B6 (H2Kb+) mothers and their progeny that received allogenic AFSC IUT (with or without fostering) at 4 weeks of age (P28; Figure 2A), and MNC were isolated using ACK lysing buffer (Lonza, New Jersey, USA). Positive control B6 (4-week-old or adult) mice were immunized with an intraperitoneal injection of approximately 2×10^{7} Balb/c (H2Kd+) cells at days 0 and 7, and MNC were harvested on day 14. Lymph node MNC from naïve, age-matched B6 mice were used as negative controls. MNC from B6 mothers, E14 injected progeny (with or without fostering), positive, and negative controls were stained with eFluor-670 dye (dilution: 1/1000; eBioscience; California, USA) and injected into the tail vein of adult Balb/c x B6 F1 mice (H2Kb+/d+). The spleen, lymph nodes, peripheral blood, BM, and thymus of the F1 mouse were harvested at 24, 48 and 72 hours; MNC were isolated and stained with anti-CD4 (clone: RM4-5; dilution 1/100; eBioscience, California, USA) and anti-H2Kd (clone: SF1-1.1; dilution: 1/100; Biolegend, California, USA) antibodies. The CD4+, H2Kd– cells were then analyzed for eFluor-670 fluorescence by FACS and the frequency of alloreactive T cells in vivo was quantified as described by Suchin et al. [25]. Briefly, the frequency of alloreactive T cells (F) was defined

as the number of cells that had divided (P^{div}), divided by the total number of cells (P^{tot}). P^{tot} was further defined as the number of cells that had successfully engrafted at 24 hours, and P^{div} was further defined as $\Sigma M_n/2^{n-1}$, where M_n represents the number of cells (M) in a given CFSE peak n, and n–1 represents the number of cell divisions that those cells have undergone. For example, cells in the second CFSE peak (n = 2) had, by definition, undergone 1 cell division, and therefore 100 cells in this peak were derived from 100 / 2^{2-1} original cells, or 50 cells. The total number of original cells that had divided (P^{div}), divided by the total number of original cells that have engrafted (P^{tot}), yielded the frequency (F).

Allo-antibody Assay

Peripheral blood was obtained by retro-orbital puncture from B6 (H2Kb+) mothers and their progeny that received allogenic AFSC IUT (with or without fostering) at P28, and serum was isolated via centrifugation. Positive control B6 (4-week-old or adult) mice were immunized with an intraperitoneal injection of approximately 2×10^7 Balb/c (H2Kd+) cells at days 0 and 7, and serum was collected on day 14. Serum from naïve, age-matched B6 mice was used as negative control. Splenocytes were isolated from adult Balb/c mice for use as allogeneic target cells, and 1×10^6 spleen cells were pre-incubated with anti-CD16/32b Fc block (Fcγ R III/II, Ly-17; 1μg per 1×10^6 cells in 100μl volume; Biolegend, California, USA) and then incubated for 45 minutes with serum from B6 mothers, E14 injected progeny (with or without fostering), positive, and negative controls at serum/splenocyte ratios of 1/100, 1/50, 1/10, 1/2, and 1/1. The cells were washed twice to remove excess serum and then incubated for 45 minutes with secondary antibody against mouse IgG (dilution 1/100; eBioscience, California, USA) to detect the presence of bound antibodies. B cells were stained with anti-CD19 (clone: 1D3; dilution 1/100; Becton Dickinson, New Jersey, USA) and excluded by gating because these cells exhibit a large amount of nonspecific binding. Cells were analyzed by FACS for mean fluorescence. Median fluorescence intensity, representing the relative concentration of alloantibodies in serum, was compared with negative control (no serum), and expressed as a fold increase.

Statistical Analysis

To achieve a statistically meaningful result, we aimed to have at least six to eight mice pups in each group, born by more than three different dams (in order to have adequate independent biological replicates). Data was tested for normality using the D'Agostino-Pearson test, and was expressed as mean ± standard error of the mean (SE) unless stated otherwise. Data was compared using unpaired student's *t*-test, one- or two-way analysis of variance (ANOVA). For multiple comparisons, p values by ANOVA were Bonferroni adjusted. In all cases, $p<0.05$ was considered statistically significant. Statistical analysis was performed using GraphPad Prism 6.0 (GraphPad Software Inc., USA).

SUPPLEMENTAL TABLES

Table S1

Antigen	Clone	Dilution	Manufacturer
CD117/c-Kit (FACS sorting)	2B8	1/100	eBioscience, USA
CD117/c-Kit (Purity check post MACS)	3C11	1/10	Miltenyi Biotec, USA
CD45	30-F11	1/100	Becton Dickinson, USA
Sca-1	D7	1/100	Becton Dickinson, USA
CD34	RAM34	1/100	Becton Dickinson, USA
H2Kb	AF6-88.5.5.3	1/100	eBioscience, USA
H2Kd	SF1-1.1	1/100	Biolegend, USA
CD3	17A2	1/100	Becton Dickinson, USA
CD4	RM4-5	1/100	eBioscience, USA
B220	RA3-6B2	1/100	Becton Dickinson, USA
CD11b	M1/70	1/100	Becton Dickinson, USA
Gr-1	RB6-8C5	1/100	Becton Dickinson, USA
Ter119	TER119	1/100	Becton Dickinson, USA
IgG	N/A	1/100	Becton Dickinson, USA
CD19	1D3	1/100	Becton Dickinson, USA

Details of antibodies used in the present experimental series.

Table S2

Gene	Primer A	Primer B	Primer C	PCR Product I	Q-PCR Product
GATA1	ATGGCAGGCTTCCATGAAAC	CACTGGCCTACTACAGAGAA	TATGGCAAGACGGCACTCTA	413 bp	297 bp
GATA2	GGTTCTGTCCATTCATCTTG	ACCACCCGATACCCACCTAT	CTAAGCAGAGAAGCAAGGCT	263 bp	152 bp
LMO2	GATAGTCTCTCCTGCACAAT	AATGTCCTCGGCCATCGAAA	CGCTACTTCCTGAAAGCCAT	253 bp	138 bp
OCT4	AGCTGATTGGCGATGTGAGT	GCGTTCTCTTTGGAAAGGTG	AGCCGACAACAATGAGAACC	286 bp	171 bp
28S	GTAGATCCTCCGGCATGTTT	TACCGGACCCTGAACAGAAT	TACCGGACCCTGAACAGAAT	128 bp	128 bp

Details of primers used in quantitative reverse-transcription polymerase chain reaction (qRT-PCR).

SUPPLEMENTAL FIGURES

Figure S1

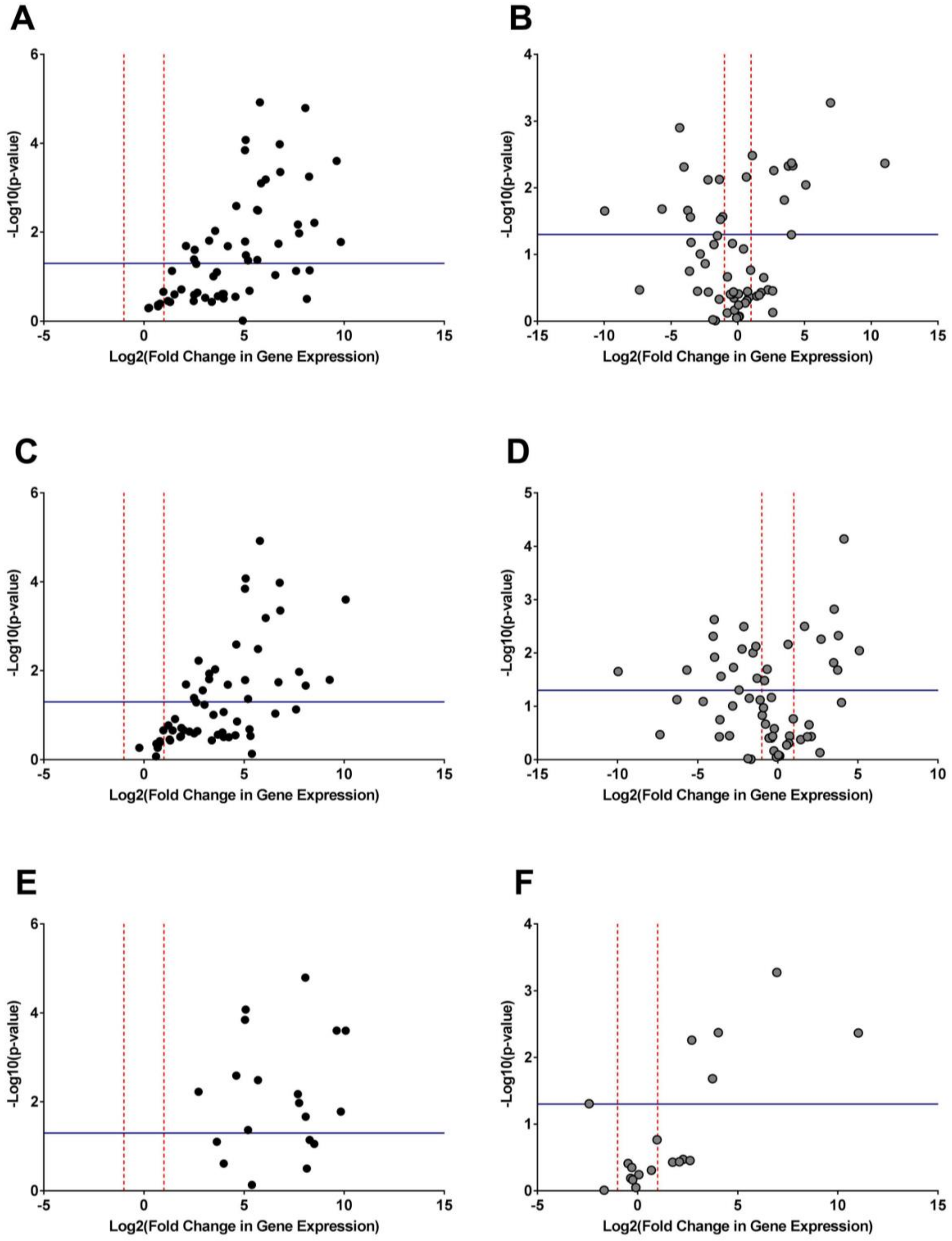

Figure S2

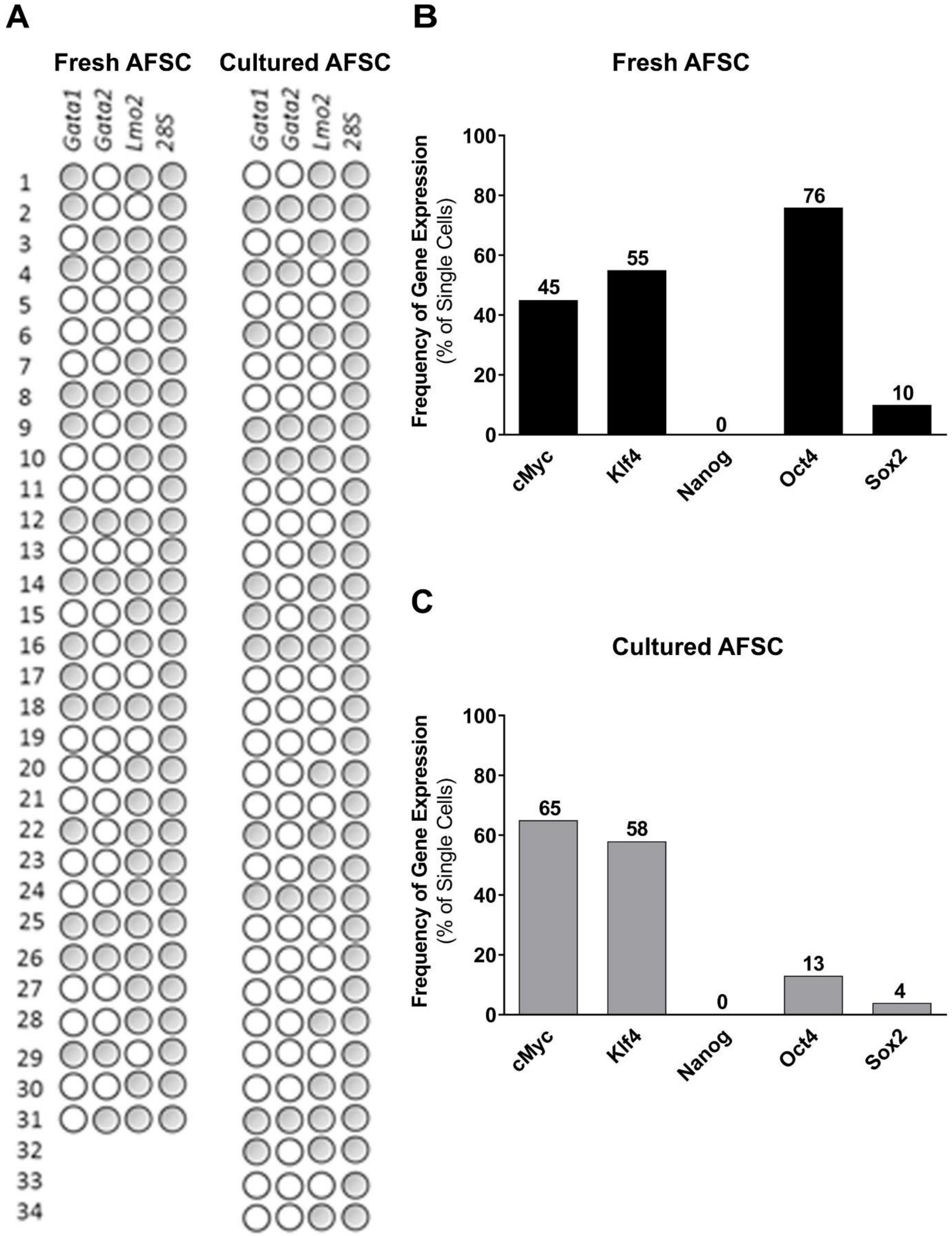
A
Fresh AFSC
Cultured AFSC
Gata1
Gata2
Lmo2
28S
1
2
3
4
5
6
7
8
9
10
11
12
13
14
15
16
17
18
19
20
21
22
23
24
25
26
27
28
29
30
31
32
33
34
B
Fresh AFSC
Frequency of Gene Expression
(% of Single Cells)
100
80
60
40
20
0
45
55
0
76
10
cMyc
Klf4
Nanog
Oct4
Sox2
C
Cultured AFSC
Frequency of Gene Expression
(% of Single Cells)
100
80
60
40
20
0
65
58
0
13
4
cMyc
Klf4
Nanog
Oct4
Sox2

Figure S3

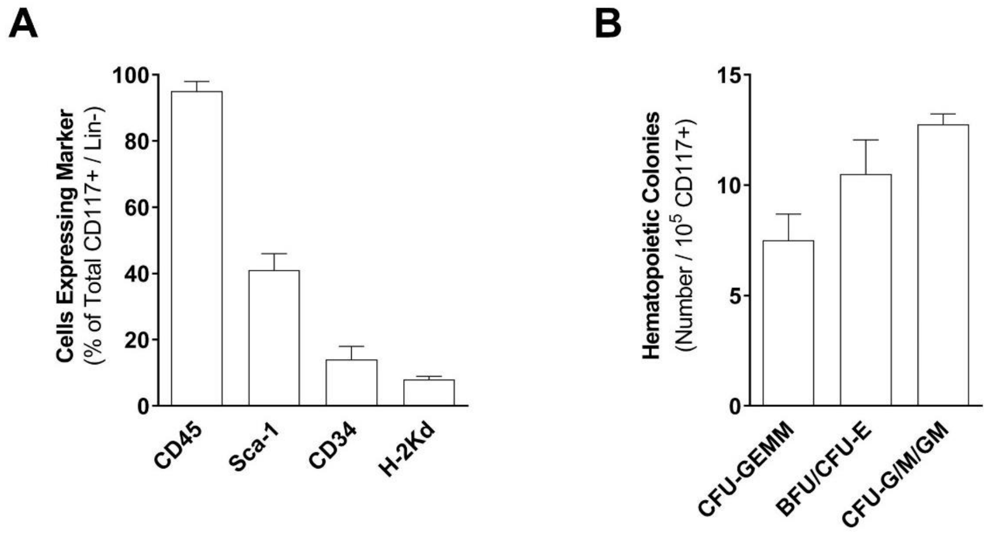
A
B
Cells Expressing Marker
(% of Total CD117+ / Lin-)
100
80
60
40
20
0
CD45
Sca-1
CD34
H-2Kd
Hematopoietic Colonies
(Number / 10^5 CD117+)
15
10
5
0
CFU-GEMM
BFU/CFU-E
CFU-G/M/GM

Figure S4

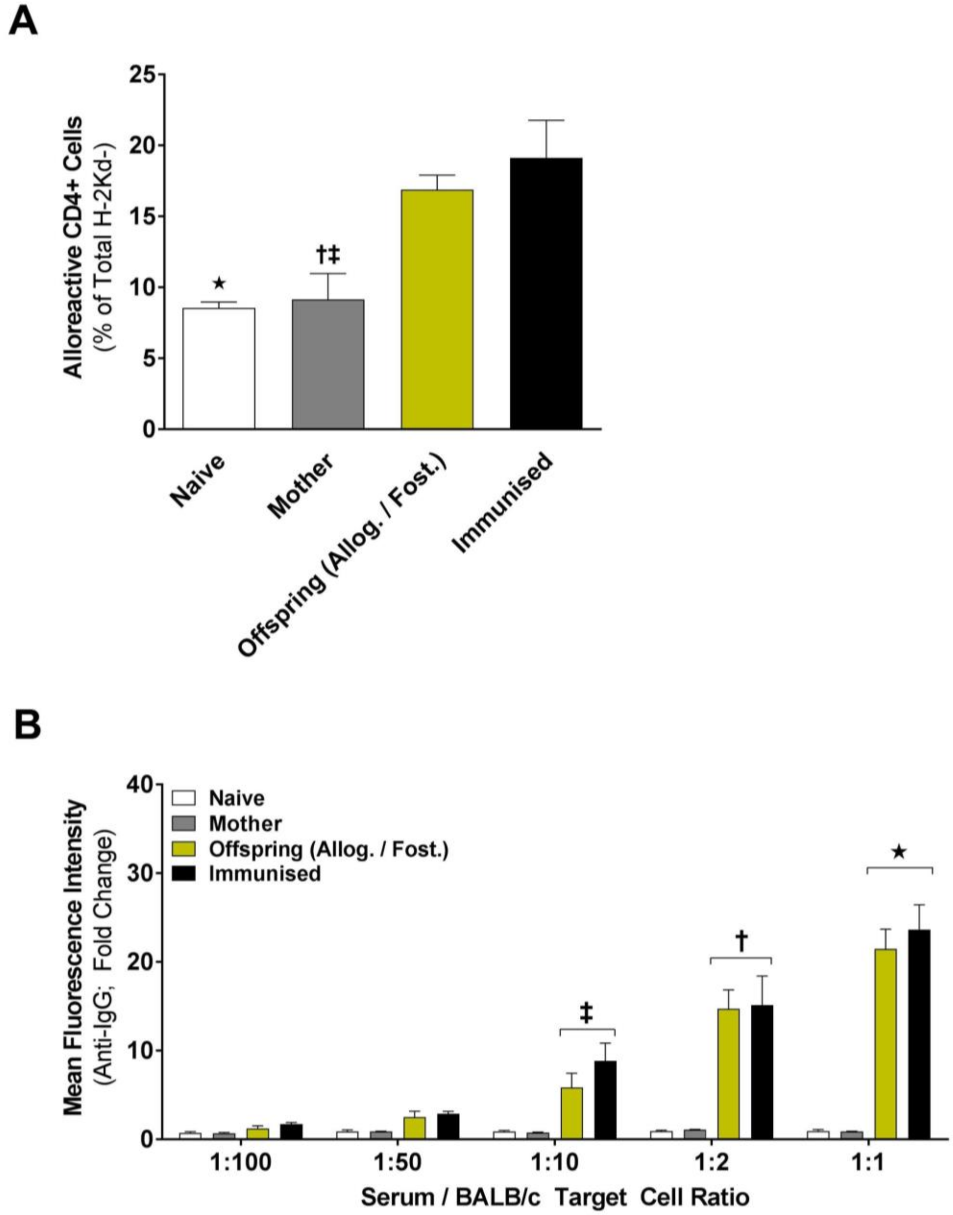
A
Alloreactive CD4+ Cells
(% of Total H-2Kd-)
25
20
15
10
5
0
★
†‡
Naive
Mother
Offspring (Allog. / Fost.)
Immunised
B
Mean Fluorescence Intensity
(Anti-IgG; Fold Change)
40
30
20
10
0
Naive
Mother
Offspring (Allog. / Fost.)
Immunised
‡
†
★
1:100
1:50
1:10
1:2
1:1
Serum / BALB/c Target Cell Ratio

Figure S5

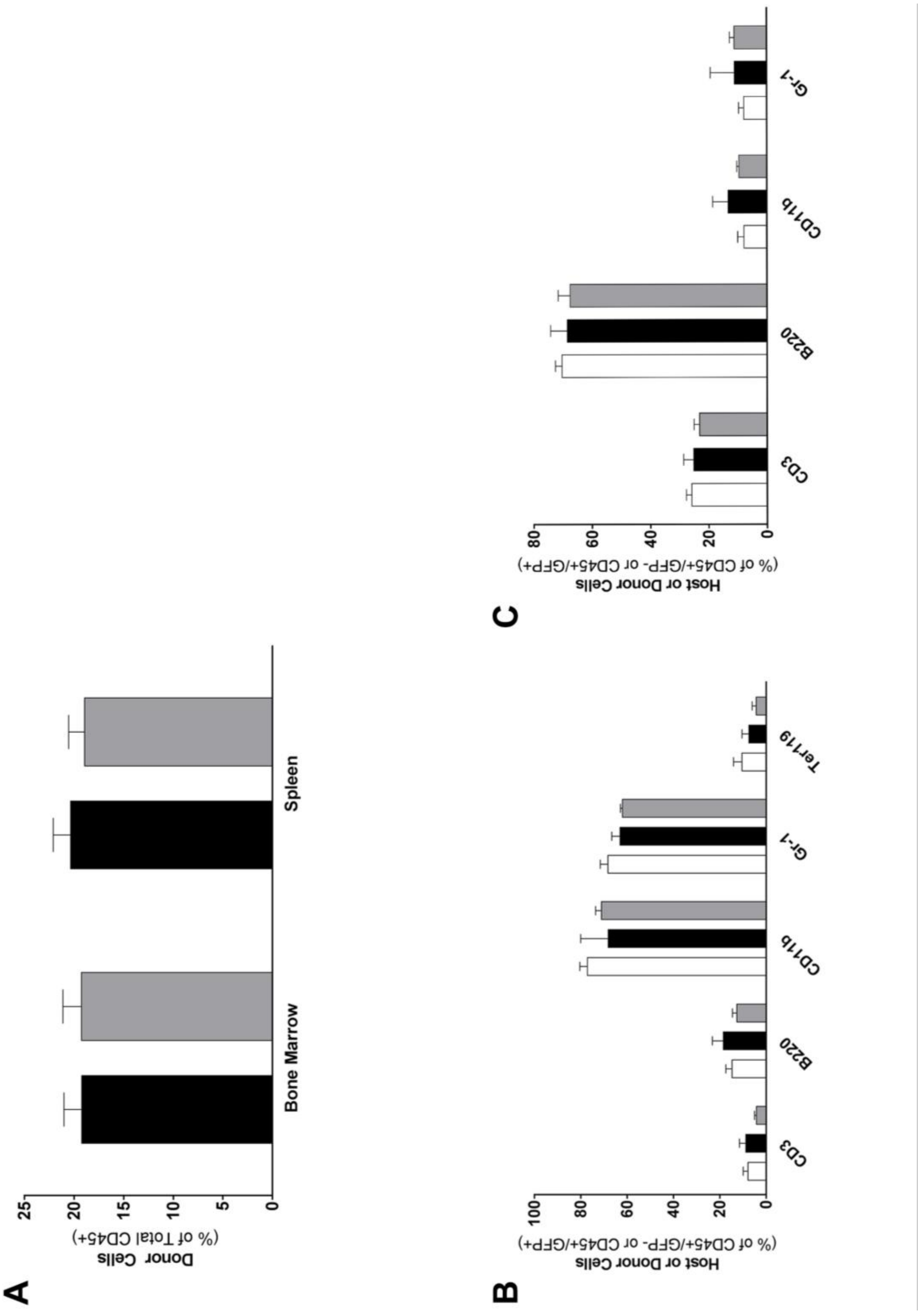
A
Donor Cells
(% of Total CD45+)
Bone Marrow
Spleen
B
Host or Donor Cells
(% of CD45+/GFP- or CD45+/GFP+)
CD3
B220
CD11b
Gr-1
Ter119
C
Host or Donor Cells
(% of CD45+/GFP- or CD45+/GFP+)
CD3
B220
CD11b
Gr-1

Figure S6

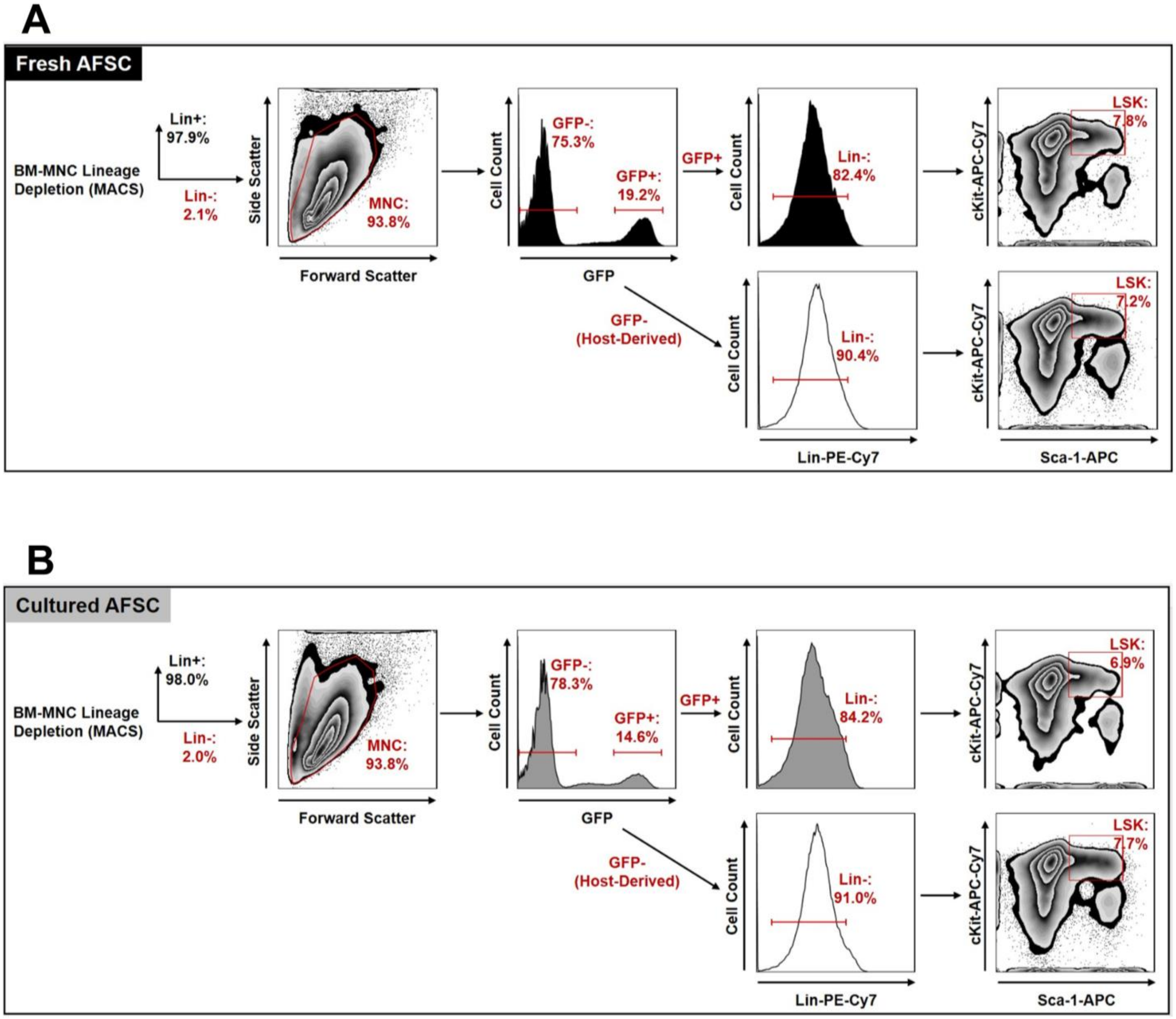

A
Fresh AFSC
BM-MNC Lineage Depletion (MACS)
Lin+: 97.9%
Lin-: 2.1%
Side Scatter
Forward Scatter
MNC: 93.8%
Cell Count
GFP
GFP-: 75.3%
GFP+: 19.2%
GFP+
GFP- (Host-Derived)
Lin-: 82.4%
Lin-: 90.4%
Lin-PE-Cy7
cKit-APC-Cy7
Sca-1-APC
LSK: 7.8%
LSK: 7.2%
B
Cultured AFSC
BM-MNC Lineage Depletion (MACS)
Lin+: 98.0%
Lin-: 2.0%
Side Scatter
Forward Scatter
MNC: 93.8%
Cell Count
GFP
GFP-: 78.3%
GFP+: 14.6%
GFP+
GFP- (Host-Derived)
Lin-: 84.2%
Lin-: 91.0%
Lin-PE-Cy7
cKit-APC-Cy7
Sca-1-APC
LSK: 6.9%
LSK: 7.7%

SUPPLEMENTARY FIGURE LEGENDS

Figure S1

Volcano plots from gene array analysis

(A) Fresh AFSC and (B) cultured AFSC are compared for haematopoiesis regulators, cell cycle regulators, signal transduction regulators, transcription factors. (C) Fresh AFSC and (D) cultured AFSC are compared for cell surface/lineage markers, blood cell differentiation/activation regulators. (E) Fresh AFSC and (F) cultured AFSC are compared for cytokines and growth factors.

Figure S2

Frequency of expression of haematopoietic and pluripotency genes in the fresh and cultured AFSC

(A) Single cells were sorted and analyzed by RT-qPCR for key regulators of haematopoiesis. Grey circles represent expression of the respective gene. Empty circles represent no expression/signal. (B) The histograms represent proportions of cells expressing the pluripotency genes c-Myc, Klf4, Oct4, Sox2, and Nanog. 28S was used as endogenous control in all cases.

[Black bars: fresh AFSC; grey bars: cultured AFSC]

Figure S3

Characterisation of freshly-isolated AFSC from Balb/c mice

C-Kit+ (CD117+) / Lineage- (Lin-) AFSC were isolated from time-dated Balb/c dams at E13. (A) The phenotype of AFSC includes CD45, Sca-1, CD34, and MHC class I (n=6); expression of these markers was similar to that observed in fresh AFSC from B6-GFP mice. (B) Absolute numbers of hematopoietic colonies formed by Balb/c AFSC cultured in semi-solid media (burst-forming and erythroid colony-forming units: BFU/CFU-E; granulocyte/macrophage colony-forming units: CFU-G/M/GM; mixed granulocyte/erythrocyte/monocyte/megakaryocyte colony-forming units (CFU-GEMM) (3 independent experiments).

Figure S4

Evidence of adaptive immune response in allogenic BM-HSC (10^4) IUT recipients

(A) Percentage of H2Kd- cells that are alloreactive CD4+ cells in F1 mice injected with lymphocytes of naïve or immunized control mice, allogenic-fostered IUT recipients, as

well as allogenic IUT recipient’s mothers. (B) Mean anti-IgG immunofluorescence intensity of mouse serum when exposed to target cells at specific ratios. Mouse serum was used from naïve or immunized control mice, allogenic-fostered IUT recipients, as well as allogenic IUT recipient’s mothers.

*[White bars: naive (no IUT)/negative control; grey bars: mother of allogenic recipients; yellow bars: offspring (allogenic-fostered); black bars: immunized/positive control; (A): * p<0.001 naive versus offspring and immunized; † p<0.01 mother versus offspring; ‡ p<0.05 mother versus immunized; (B): * p<0.0001, † p<0.001, and ‡ p<0.01 offspring and immunized versus naive and mother]*

Figure S5

Long-term multi-lineage hematopoietic engraftment post IUT of fresh and cultured AFSC

(A) 6-month engraftment levels of fresh and cultured AFSC in bone marrow and spleen. (B) Multilineage analysis of the recipient bone marrow at 6 months post-IUT of fresh and cultured AFSC. (C) Multilineage analysis of the recipient spleen at 6 months post-IUT of fresh and cultured AFSC.

[Black bars: fresh AFSC/fresh AFSC-derived; grey bars: cultured AFSC/cultured AFSC-derived; white bars: host-derived]

Figure S6

Isolation of bone marrow hematopoietic stem cells by FACS sorting

Bone marrow mononuclear cells (BM-MNC) were isolated from 6-month old B6 recipient animals (GFP-) that underwent in utero transplantation (IUT; at E14) of fresh or cultured AFSC from B6-GFP mice. After lineage depletion (magnetic separation; MACS), remaining lineage negative (Lin-) MNC were FACS-sorted to isolate hematopoietic stem cells (CD117+, Sca-1+, Lin-; BM-HSC or LSK). Donor-derived (fresh or cultured AFSC-derived) BM-HSC/LSK were GFP+ and were isolated separately from host-derived (GFP-) cells. Isolated BM-HSC were used for subsequent gene array analysis. (A) Representative FACS gating plot for BM-HSC/LSK isolation 6 months after IUT of fresh AFSC at E14 (6 independent experiments). MACS lineage depletion removed 97.9% of the total BM-MNC (2.1% of total BM-MNC used for FACS sorting). The donor/fresh AFSC-derived/GFP+ cell chimerism within the total Lin- cells was 19.2%. We calculated that the proportion of donor/fresh AFSC-derived/GFP+ HSC within the total donor/fresh AFSC-derived/GFP+ mononuclear cells was 0.13% ([2.1/100] * [93.8/100] * [82.4/100] * [7.8/100] * 100), and the proportion of host-derived/GFP- HSC within the total host-derived/GFP-mononuclear cells was 0.12% ([2.1/100] * [93.8/100] * [90.4/100] * [7.2/100] * 100). (B) Representative FACS gating plot for BM-HSC/LSK isolation 6 months after IUT of cultured AFSC at E14 (6 independent experiments). MACS lineage depletion removed

98.0% of the total BM-MNC (2.0% of total BM-MNC used for FACS sorting). The donor/cultured AFSC-derived/GFP+ cell chimerism within the total Lin- cells was 14.6%.We calculated that the proportion of donor/cultured AFSC-derived/GFP+ HSC within the total donor/cultured AFSC-derived/GFP+ mononuclear cells was 0.10% ([2.0/100] * [91.6/100] * [84.2/100] * [6.9/100] * 100), and the proportion of host-derived/GFP- HSC within the total host-derived/GFP- mononuclear cells was 0.13% ([2.0/100] * [91.6/100] * [91.0/100] * [7.7/100] * 100).
